# Supplementary material for: Senescent endothelial cells promote pathogenic neutrophil trafficking in inflamed tissues
Source: EMBO Rep. 2024 Jun 25;25(9):10. doi: 10.1038/s44319-024-00182-x (PMC11387759; doi:10.1038/s44319-024-00182-x)
Supplement: Supplementary file 2 — Movie EV1 [file 44319_2024_182_MOESM2_ESM.zip › Readme Movie EV1.docx]

**Movie EV1. Correlation between tdTmt positive and Progerin-expressing endothelial cells.** Video illustrating the vascular system of the cremaster muscle in *Tie2-Cre:Lmna^LCS/LCS^;Rosa26^tdTomato/+^* mice that were used in this study to display tdTomato- (empty) or tdTomato+ (Magenta) ECs by confocal microscopy. EC junctions were immunostained *in vivo* with a fluorescently-labelled anti-PECAM-1 mAb (blue) and fixed cremaster muscles were subsequently immunostained with our novel anti-mouse-Progerin pAb (white). The movie sweeps through the cremasteric vascular system, highlighting the mosaic expression of tdTomato expression within ECs. A zoom-in view of a selected region, where both tdTomato-positive and -negative ECs were present, was utilized to highlight the complete correlation between tdTomato and progerin expression in ECs.
