# Supplementary material for: Senescent endothelial cells promote pathogenic neutrophil trafficking in inflamed tissues
Source: EMBO Rep. 2024 Jun 25;25(9):10. doi: 10.1038/s44319-024-00182-x (PMC11387759; doi:10.1038/s44319-024-00182-x)
Supplement: Supplementary file 3 — Movie EV2 [file 44319_2024_182_MOESM3_ESM.zip › Readme Movie EV2.docx]

**Movie EV 2. Dynamics of neutrophil interaction with tdTmt positive and tdTmt negative endothelial cells.**

The confocal IVM movie captures an IL-1β-stimulated cremasteric venule of a *Tie2-Cre:Lmna^LCS/LCS^;Rosa26^tdTomato/+^;Lyz2-EFGP-ki* mouse exhibiting GFP^bright^ neutrophils and tdTomato- (empty) or tdTomato+ (Magenta) ECs by confocal microscopy. EC junctions were immunostained *in vivo* with a fluorescently-labelled anti-PECAM-1 mAb (blue). The video shows the interaction between neutrophils and tdTomato-positive and -negative ECs over a period of 20 minutes. For enhanced clarity, the recorded venular segment is presented in two modes: the upper panel displays neutrophils (green) and ECs (blue), while the lower panel video depicts ECs (blue) and tdTomato (magenta). Together, the movies illustrate increased attachment of neutrophils to tdTomato-rich regions of a post-capillary venule. This observation is further highlighted via zoomed-in areas.
