# Supplementary material for: Senescent endothelial cells promote pathogenic neutrophil trafficking in inflamed tissues
Source: EMBO Rep. 2024 Jun 25;25(9):10. doi: 10.1038/s44319-024-00182-x (PMC11387759; doi:10.1038/s44319-024-00182-x)
Supplement: Supplementary file 4 — Movie EV3 [file 44319_2024_182_MOESM4_ESM.zip › Readme Movie EV3.docx]

**Movie EV 3. Exemplar neutrophil crawling over tdTmt positive and tdTmt negative endothelial cells.**

The confocal IVM movie captures an IL-1β-stimulated cremasteric venule of a *Tie2-Cre:Lmna^LCS/LCS^;Rosa26^tdTomato/+^;Lyz2-EFGP-ki* mouse exhibiting GFPbright neutrophils and tdTomato- (empty) or tdTomato+ (magenta) ECs by confocal microscopy. EC junctions were immunostained *in vivo* with a fluorescently-labelled anti-PECAM-1 mAb (blue). The movie isolates and tracks a single neutrophil that first crawls on a tdTomato-positive EC at a reduced 13

speed (~3.8 μm/min) and then, at a faster speed (~5.4 μm/min) on a tdTomato-negative ECs over a period of 22 minutes.
