# Supplementary figures and images for: Senescent endothelial cells promote pathogenic neutrophil trafficking in inflamed tissues
Source: EMBO Rep. 2024 Jun 25;25(9):10. doi: 10.1038/s44319-024-00182-x (PMC11387759; doi:10.1038/s44319-024-00182-x)

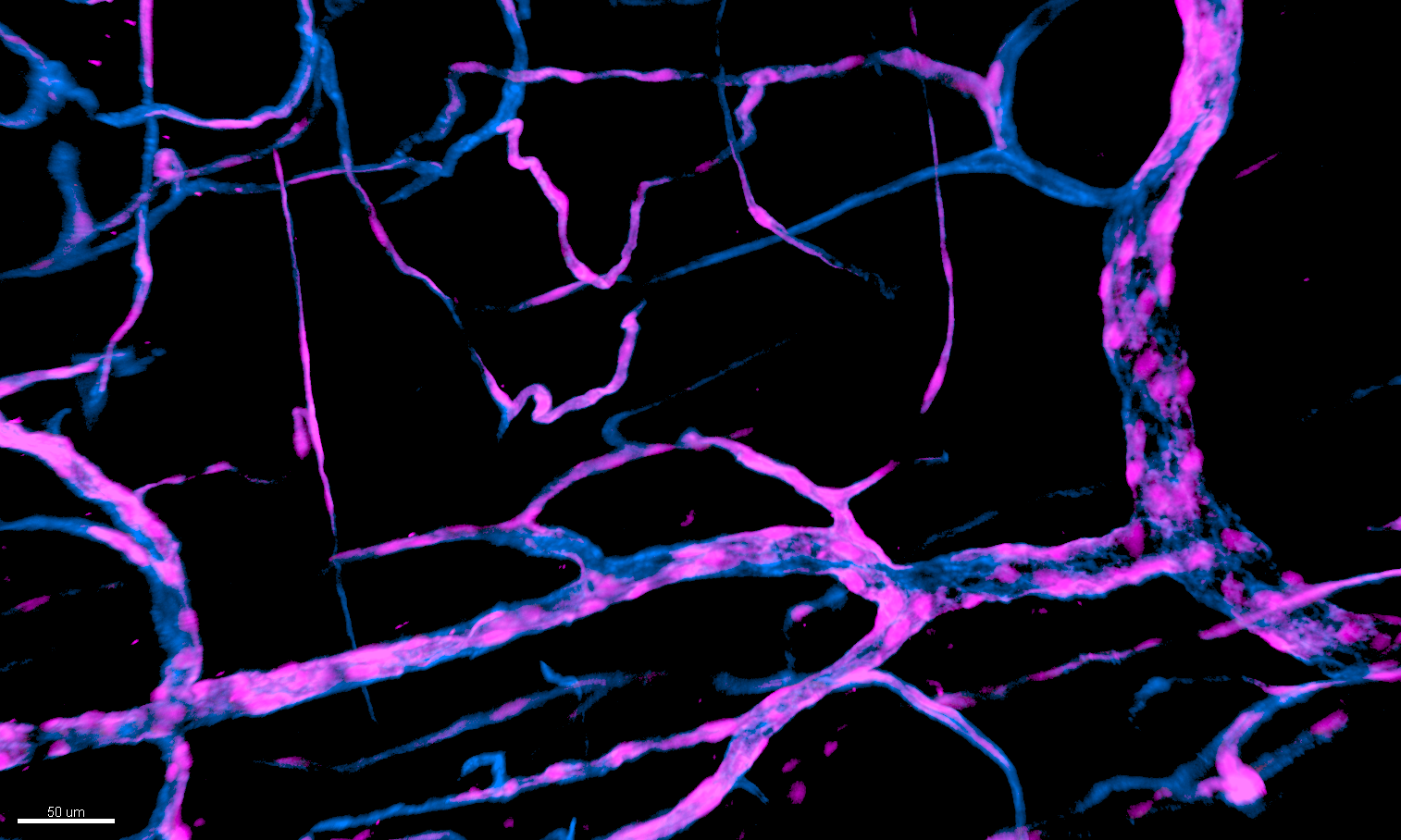

Supplement: Supplementary file 5 — Source data Fig. 1 [file 44319_2024_182_MOESM5_ESM.zip › 1B.tif]

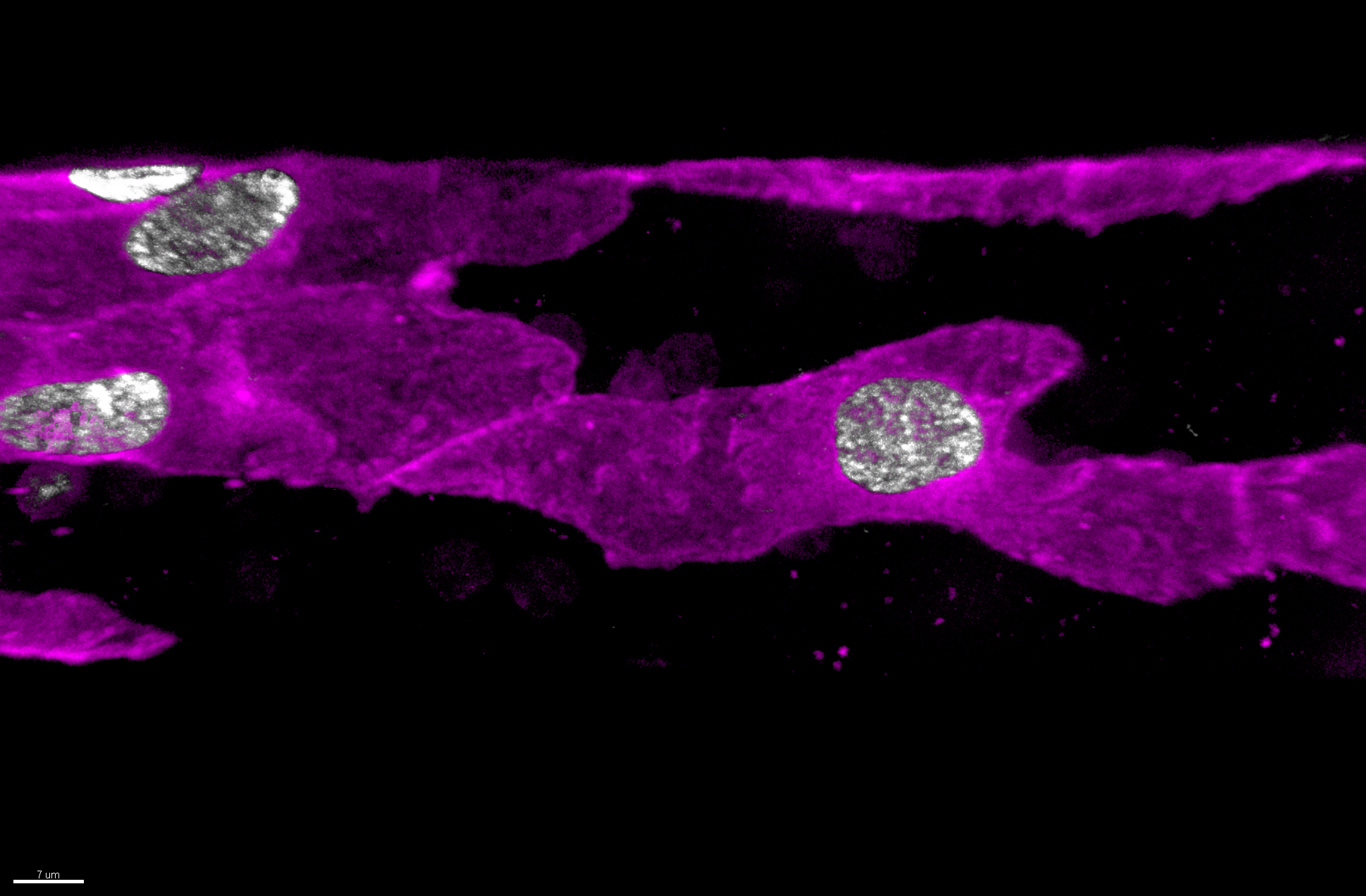

Supplement: Supplementary file 5 — Source data Fig. 1 [file 44319_2024_182_MOESM5_ESM.zip › 1D/1D left side.tif]

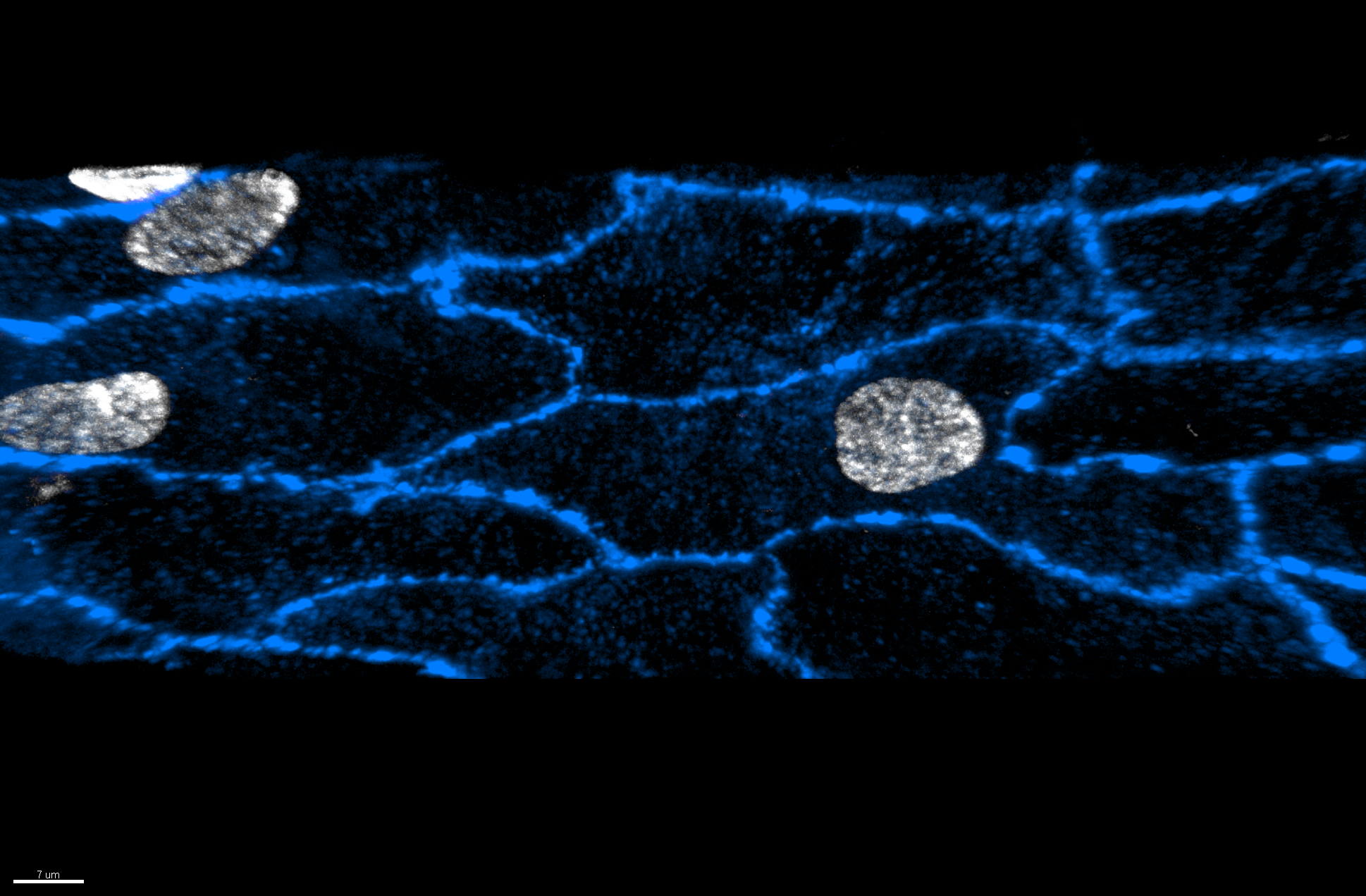

Supplement: Supplementary file 5 — Source data Fig. 1 [file 44319_2024_182_MOESM5_ESM.zip › 1D/1D right side.tif]

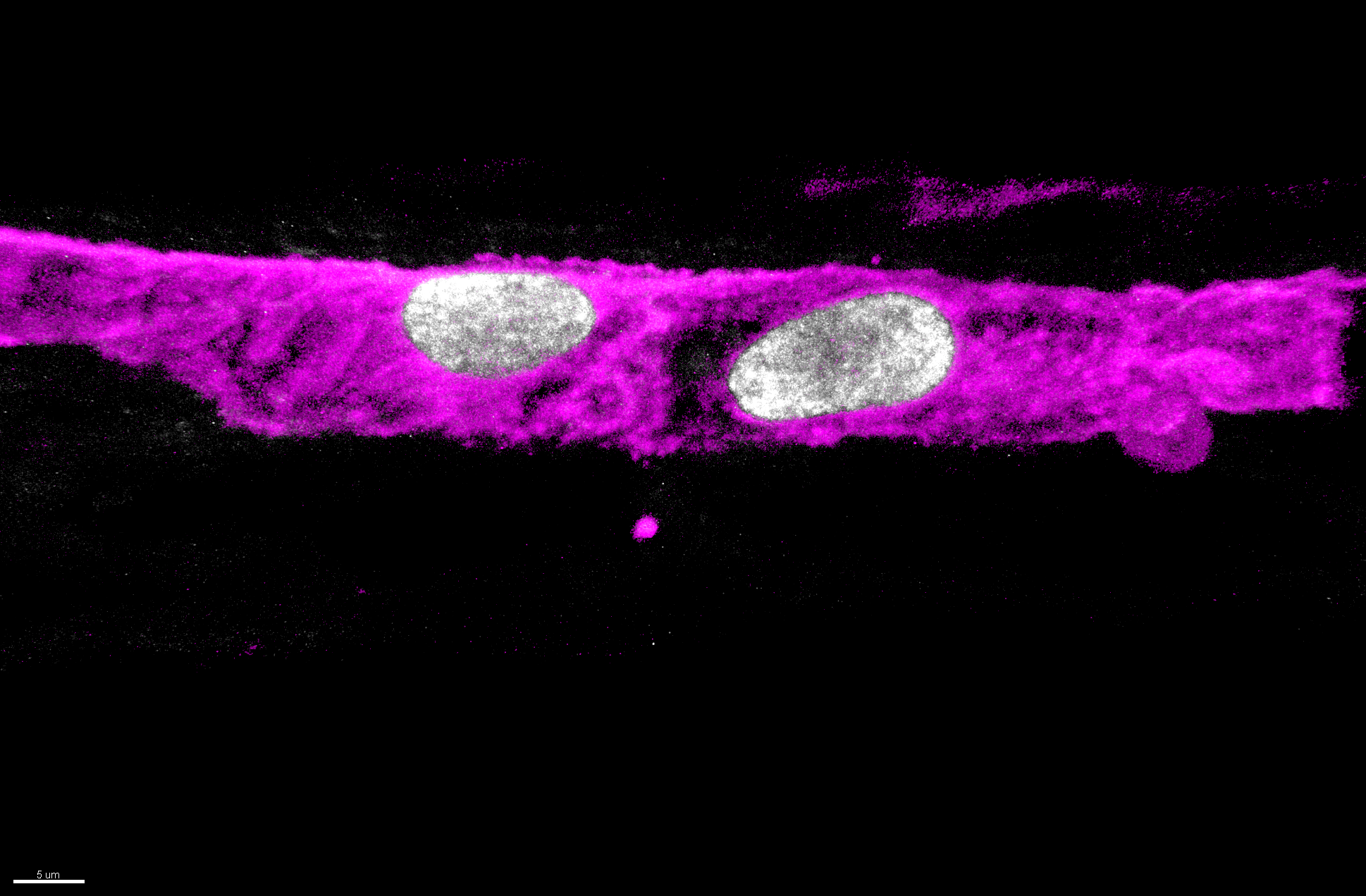

Supplement: Supplementary file 5 — Source data Fig. 1 [file 44319_2024_182_MOESM5_ESM.zip › 1F/1F ear left side.tif]

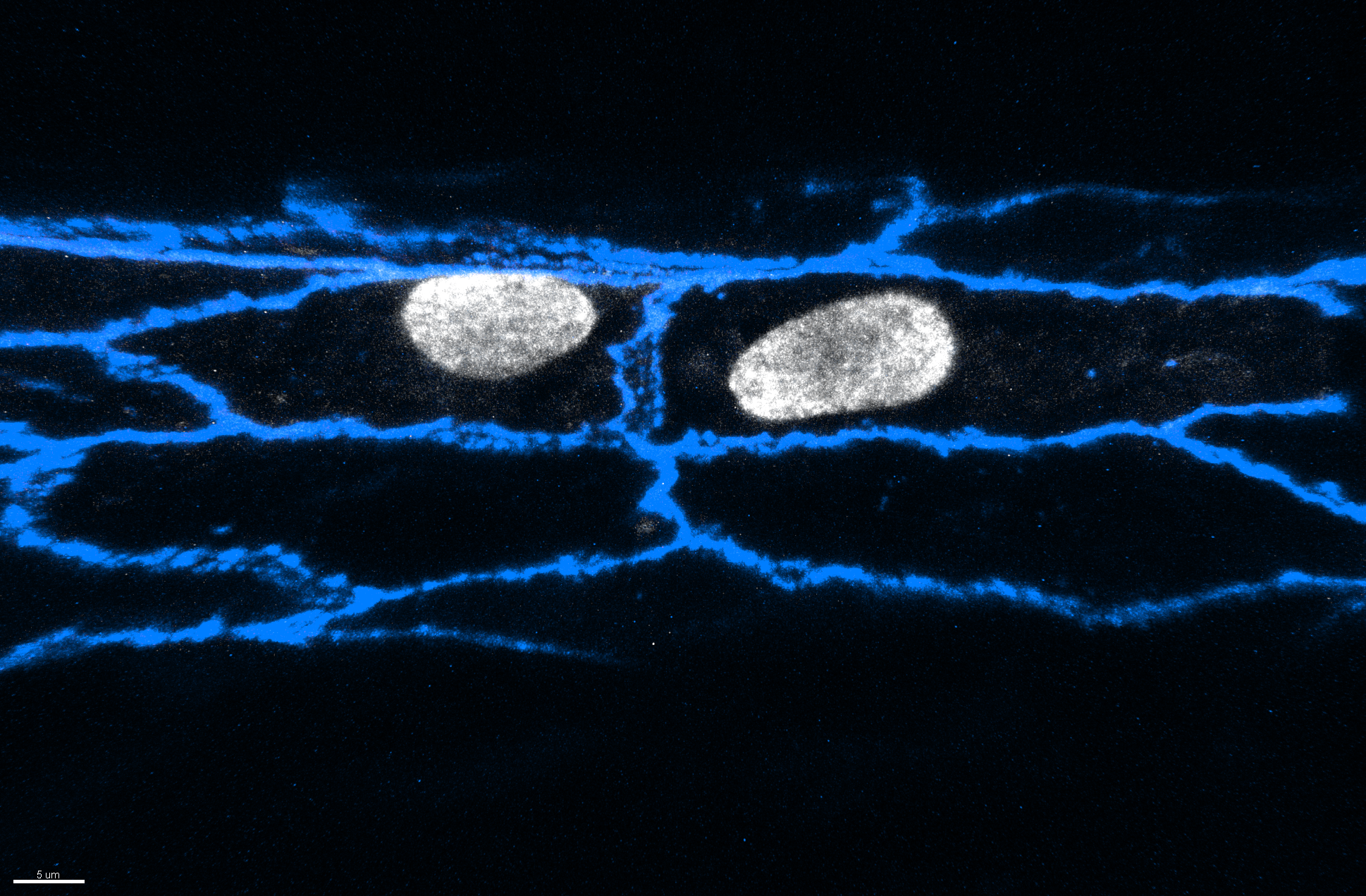

Supplement: Supplementary file 5 — Source data Fig. 1 [file 44319_2024_182_MOESM5_ESM.zip › 1F/1F ear right side.tif]

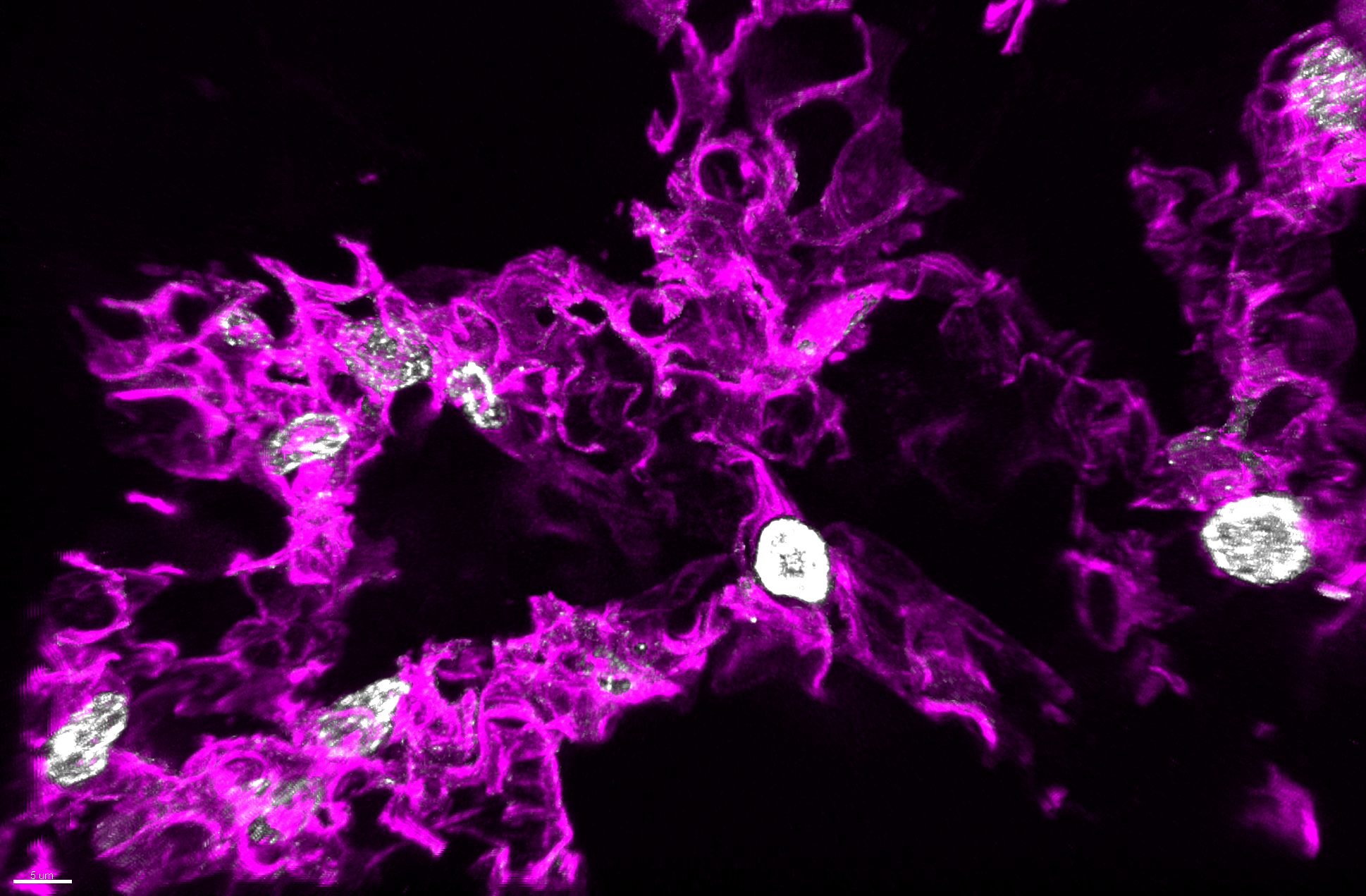

Supplement: Supplementary file 5 — Source data Fig. 1 [file 44319_2024_182_MOESM5_ESM.zip › 1F/1F lung left side.tif]

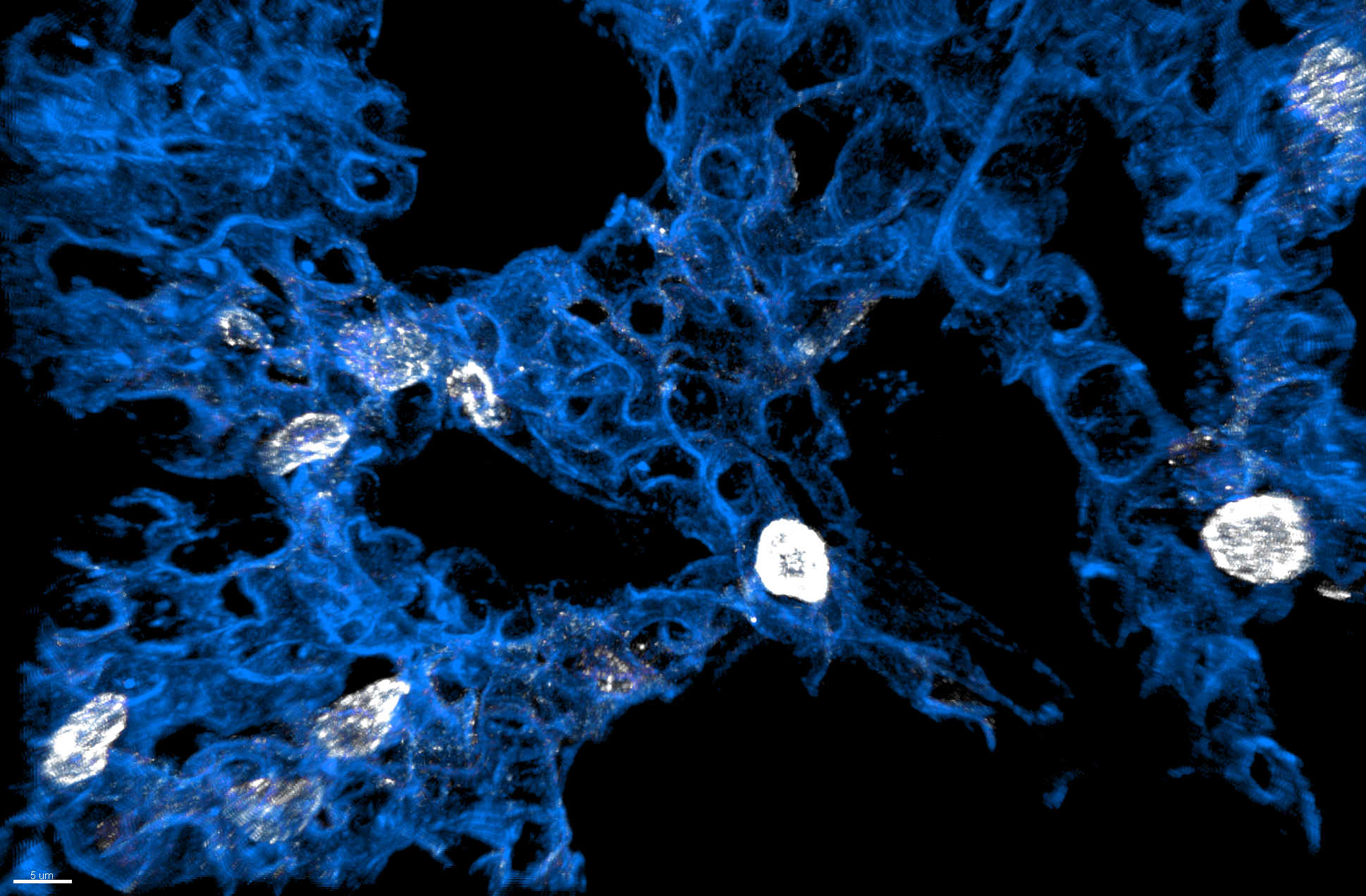

Supplement: Supplementary file 5 — Source data Fig. 1 [file 44319_2024_182_MOESM5_ESM.zip › 1F/1F lung right side.tif]

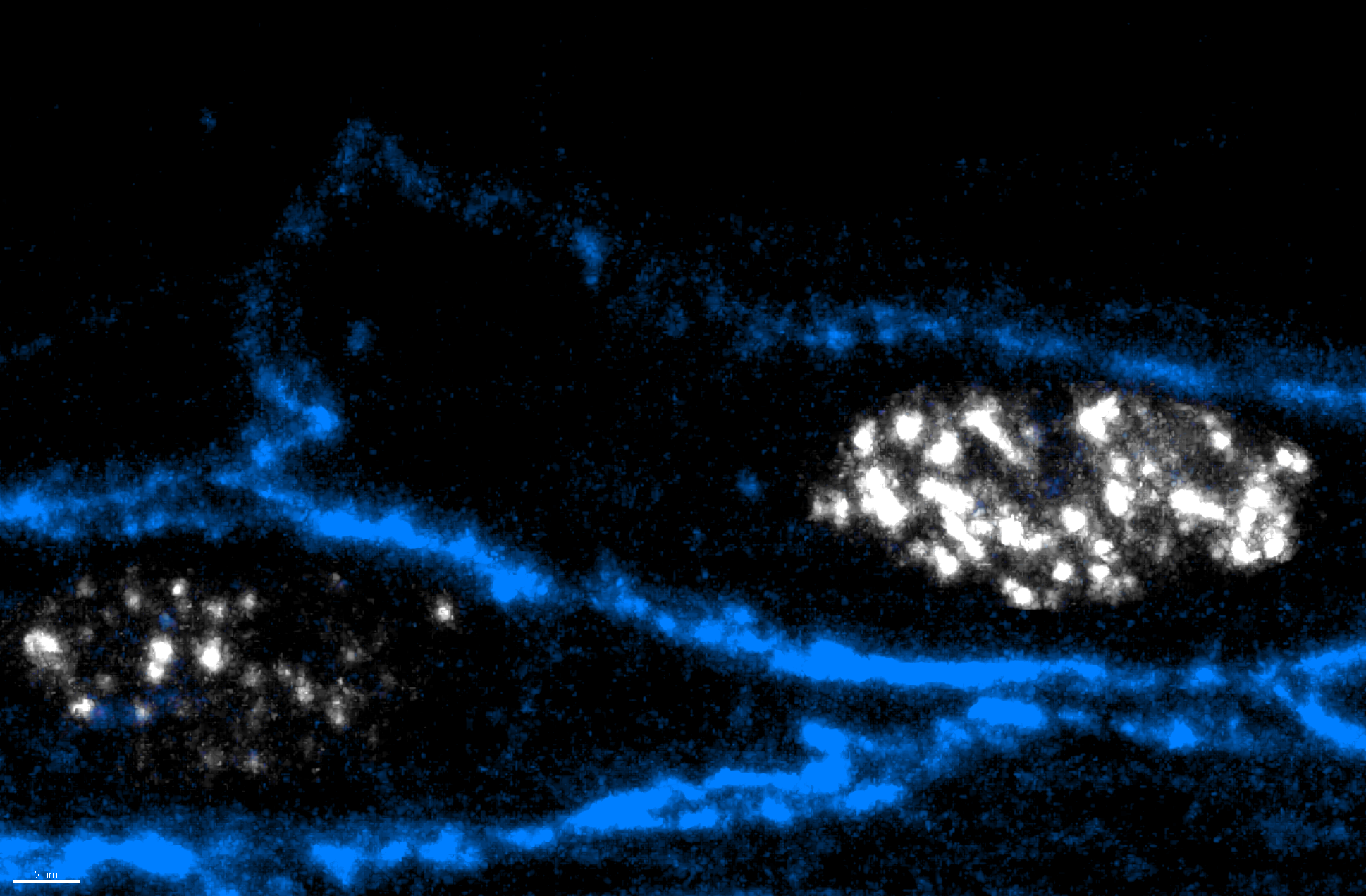

Supplement: Supplementary file 6 — Source data Fig. 2 [file 44319_2024_182_MOESM6_ESM.zip › 2A/2A bottom.tif]

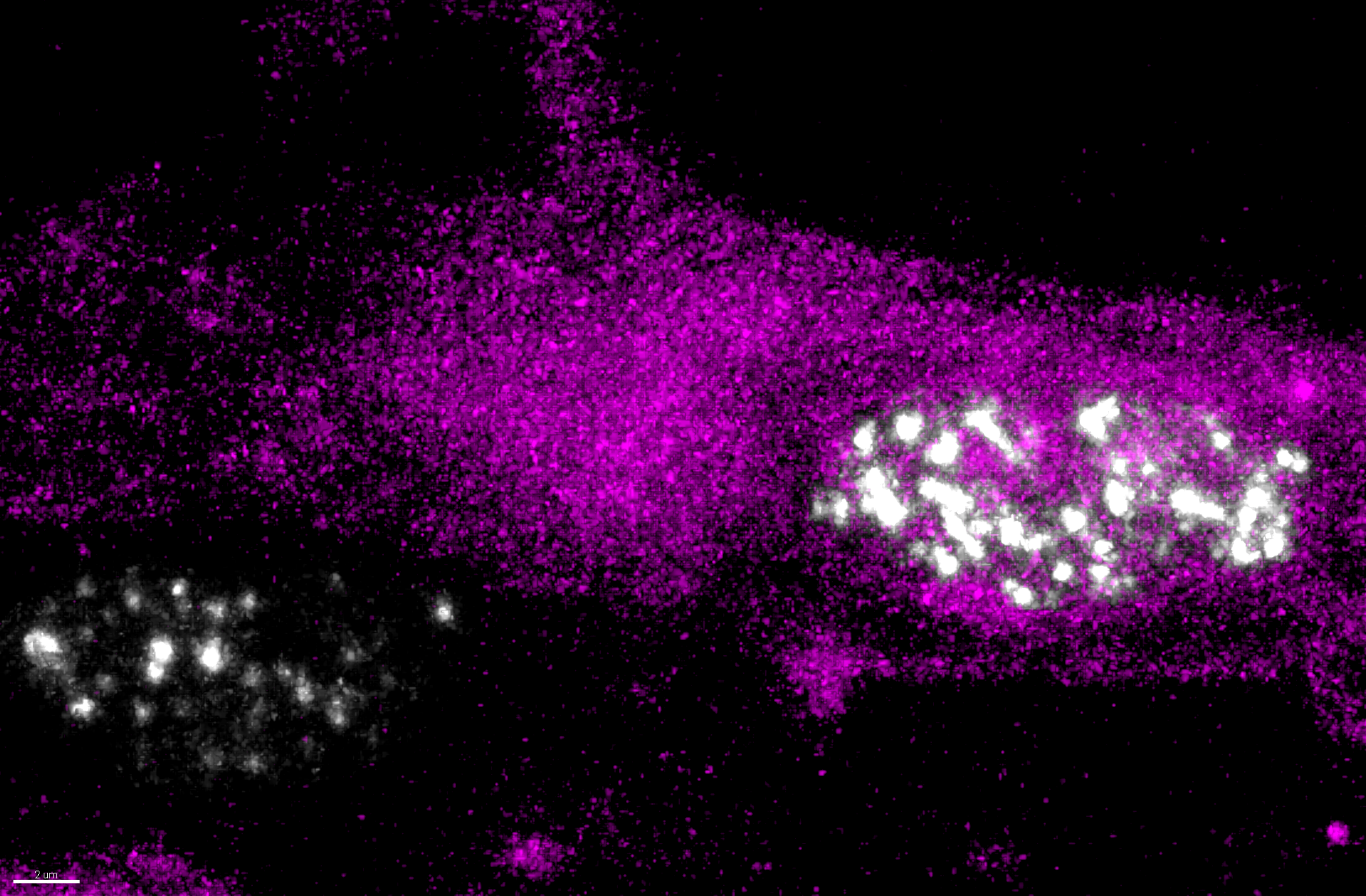

Supplement: Supplementary file 6 — Source data Fig. 2 [file 44319_2024_182_MOESM6_ESM.zip › 2A/2A top.tif]

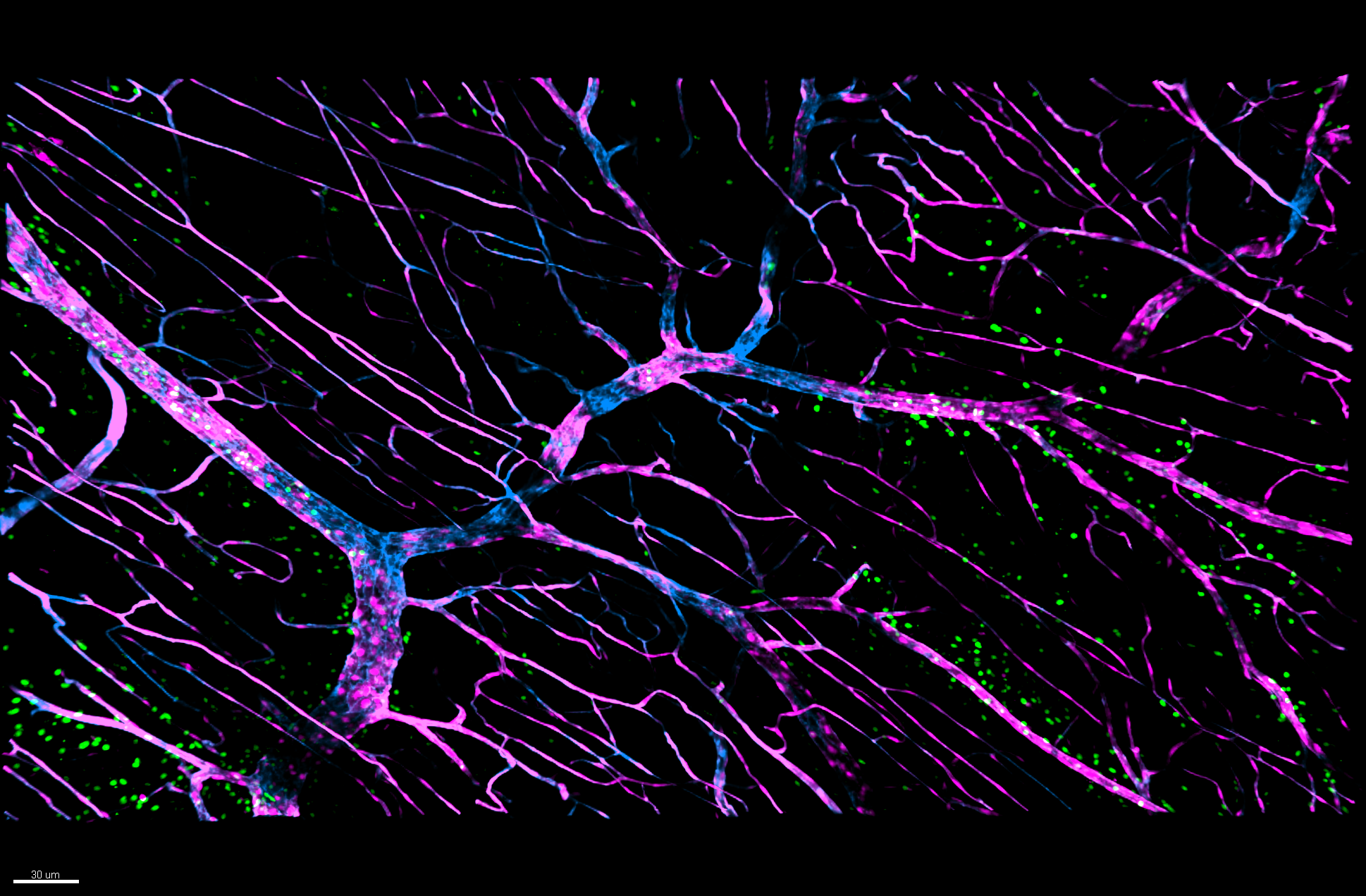

Supplement: Supplementary file 7 — Source data Fig. 3 [file 44319_2024_182_MOESM7_ESM.zip › 3A/3A full tile scan.tif]

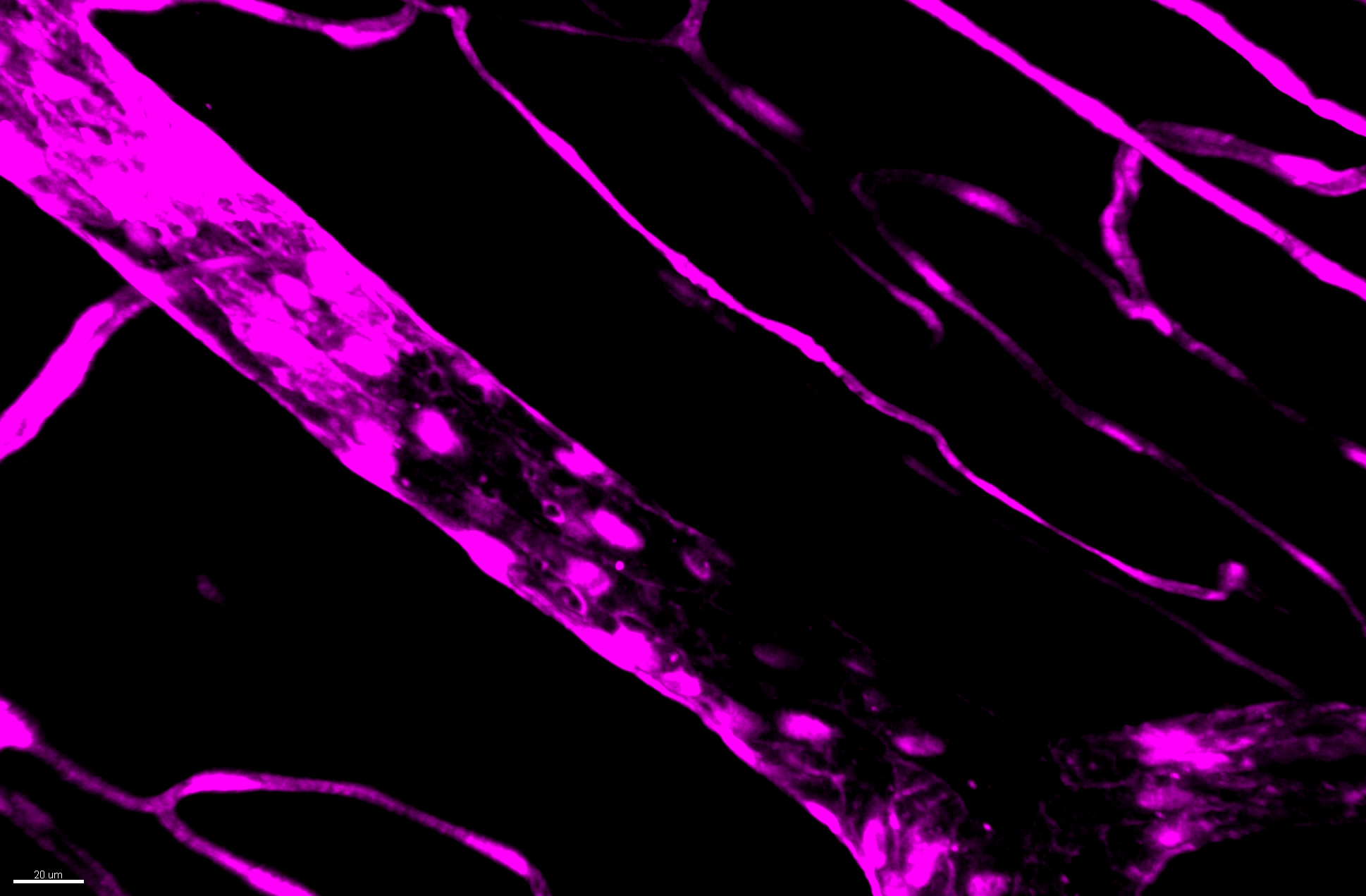

Supplement: Supplementary file 7 — Source data Fig. 3 [file 44319_2024_182_MOESM7_ESM.zip › 3A/3A section 1 center.tif]

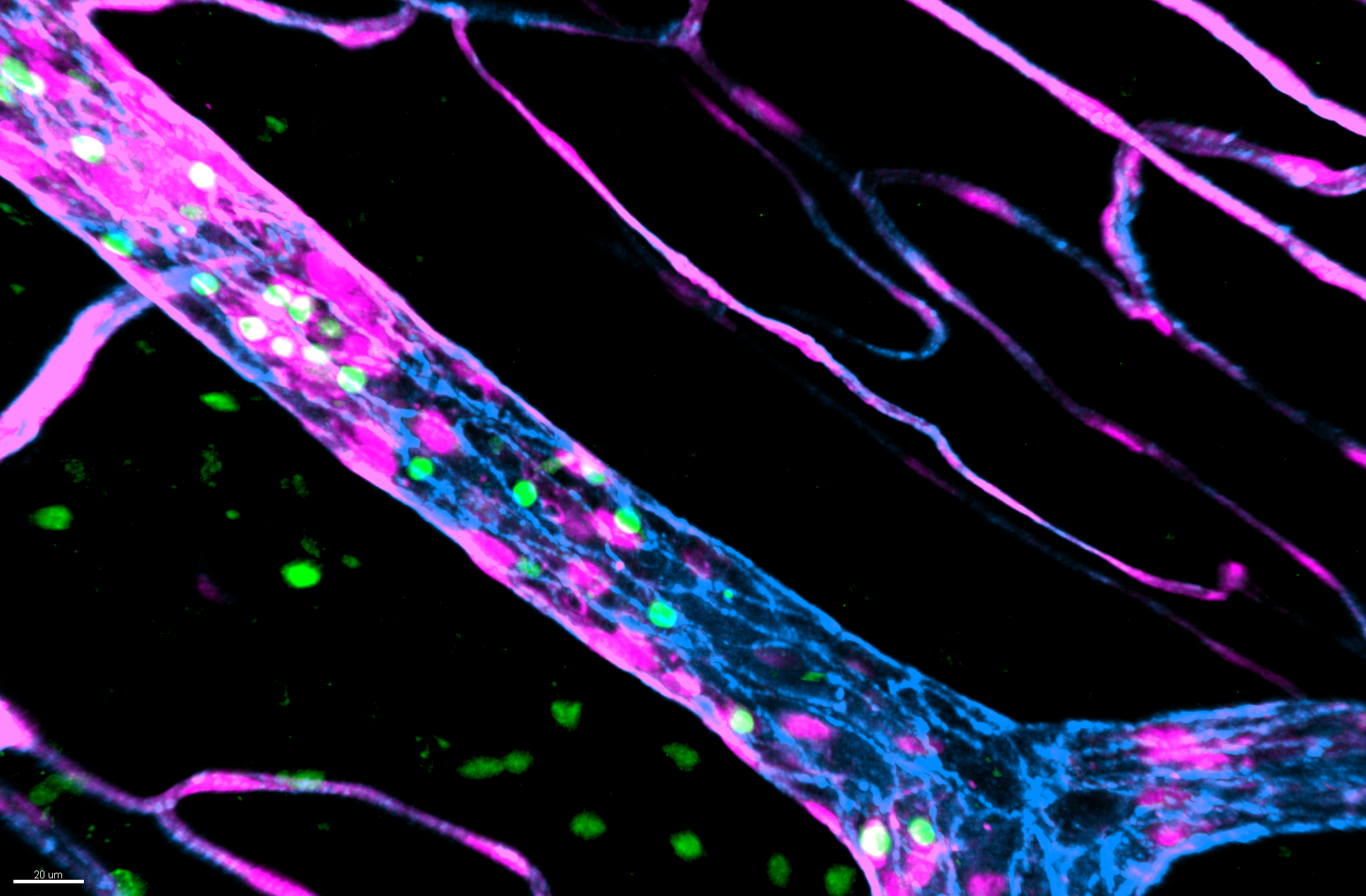

Supplement: Supplementary file 7 — Source data Fig. 3 [file 44319_2024_182_MOESM7_ESM.zip › 3A/3A section 1 left.tif]

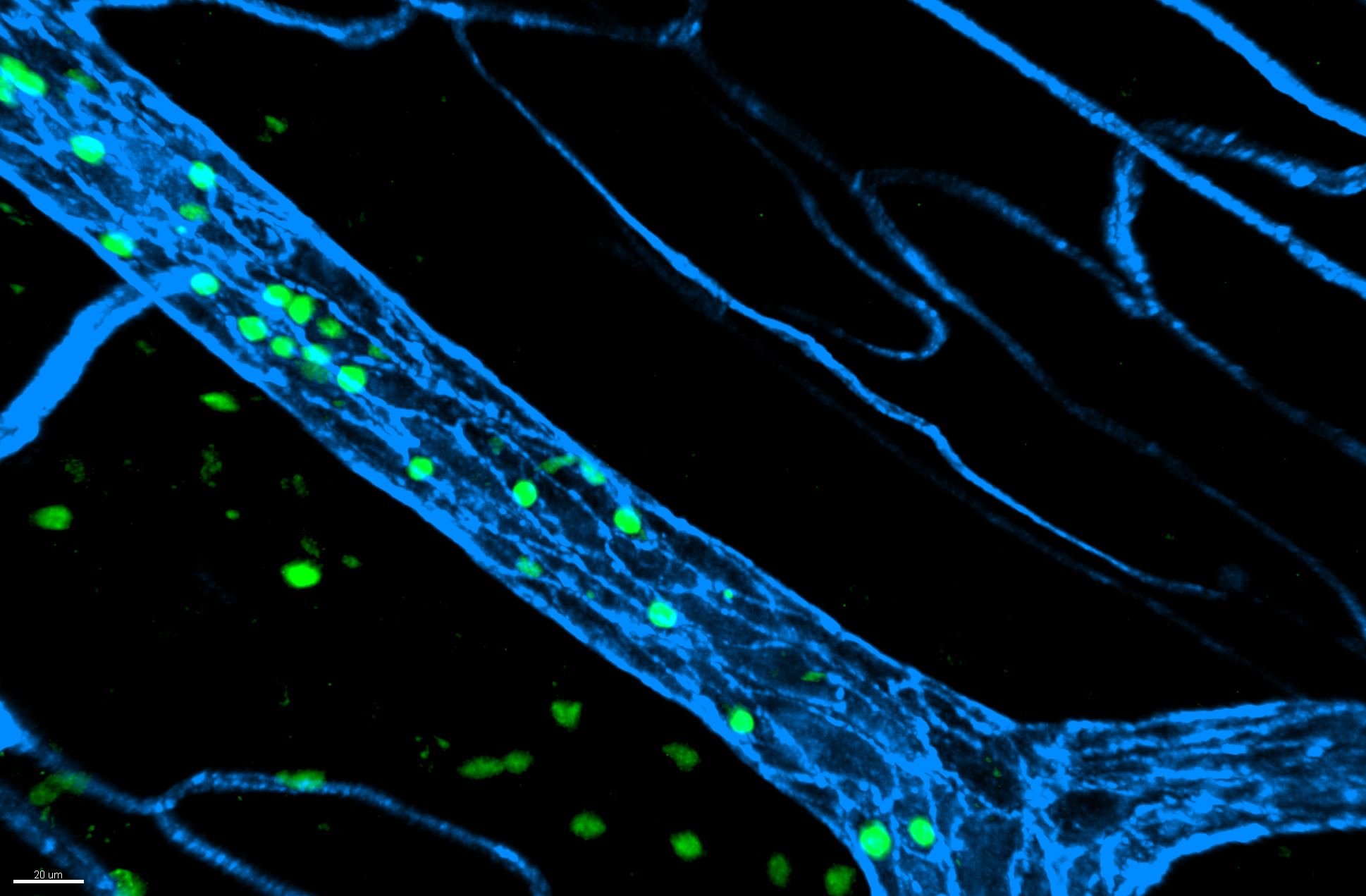

Supplement: Supplementary file 7 — Source data Fig. 3 [file 44319_2024_182_MOESM7_ESM.zip › 3A/3A section 1 right.tif]

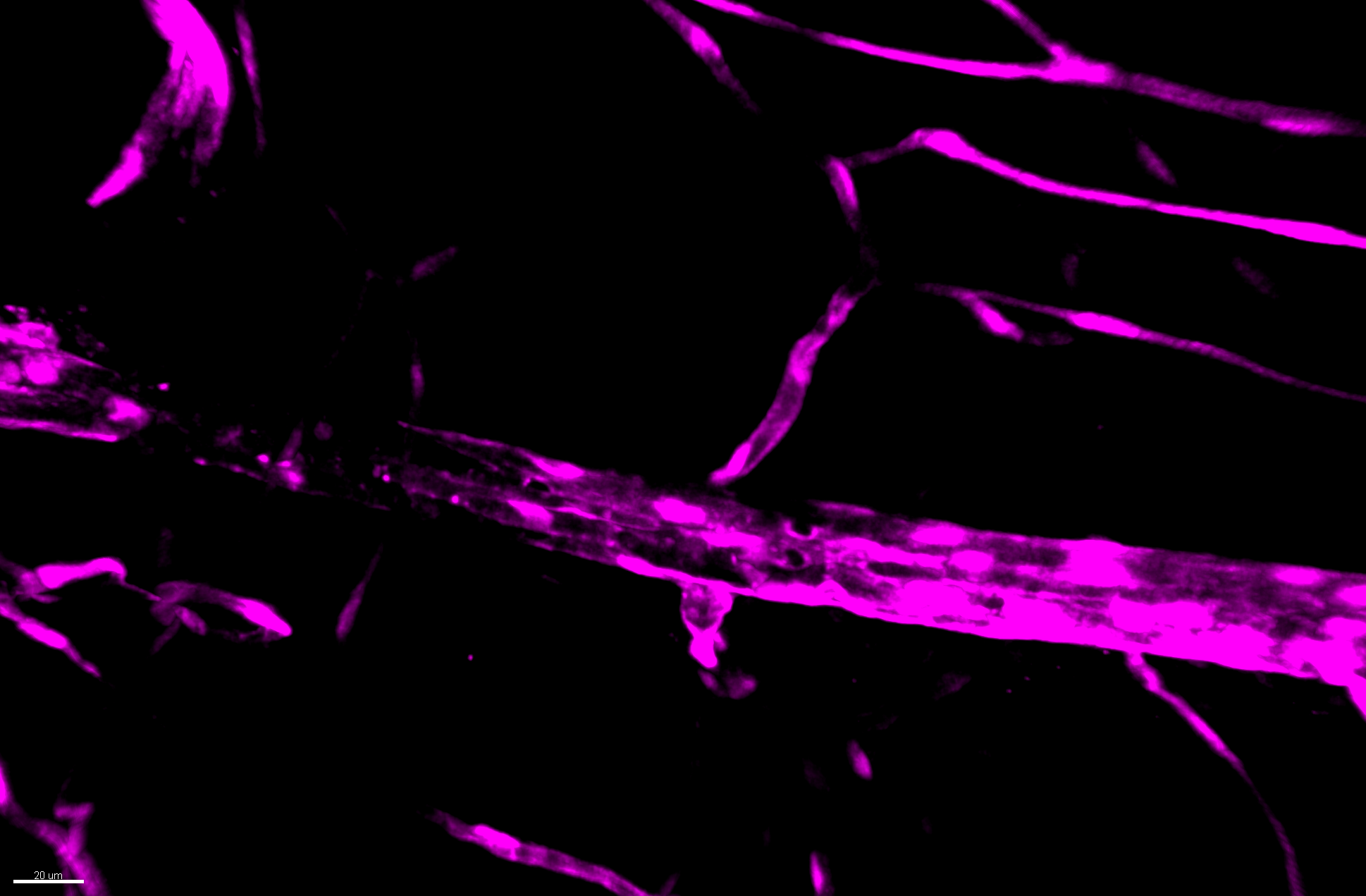

Supplement: Supplementary file 7 — Source data Fig. 3 [file 44319_2024_182_MOESM7_ESM.zip › 3A/3A section 2 center.tif]

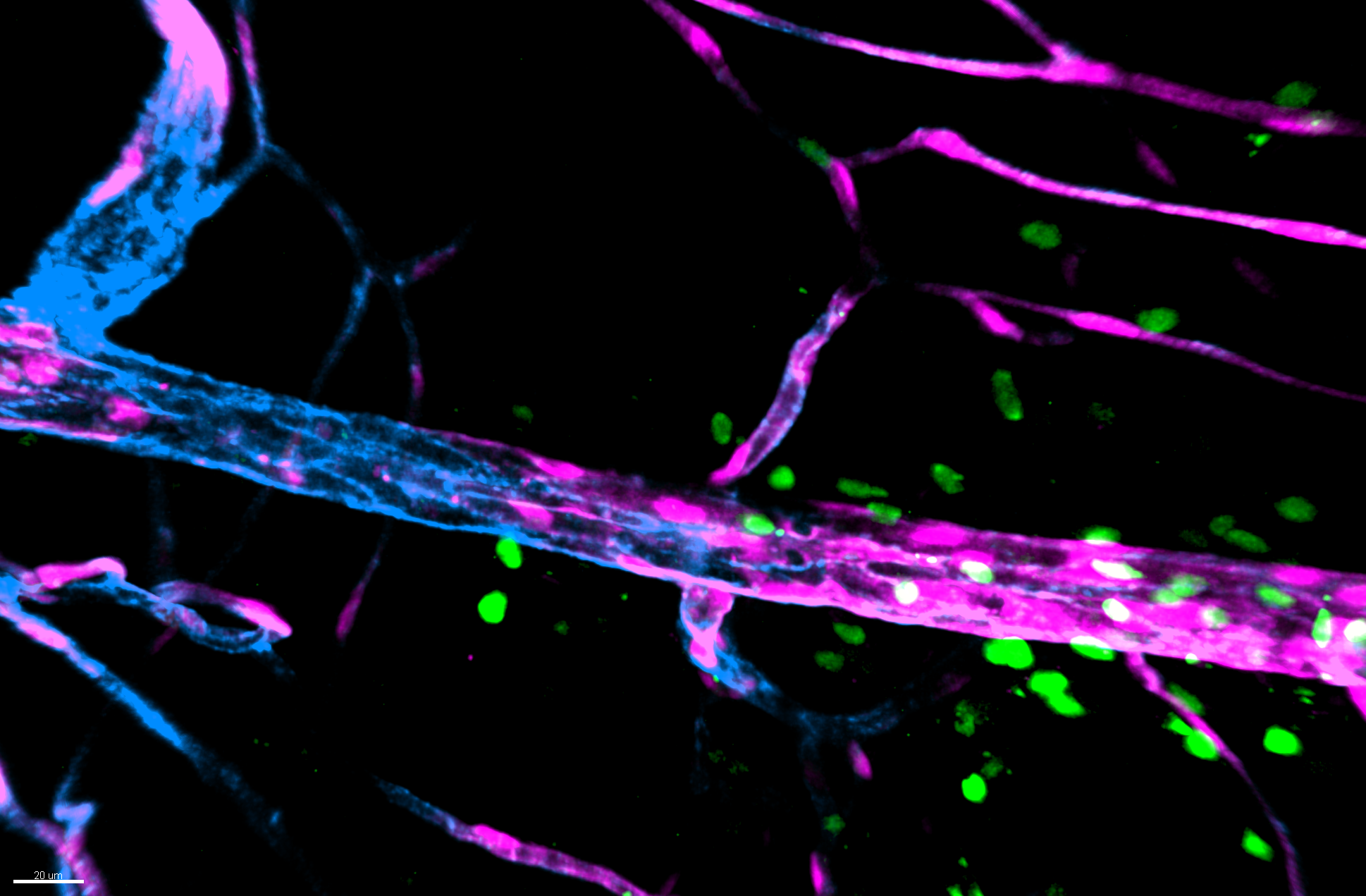

Supplement: Supplementary file 7 — Source data Fig. 3 [file 44319_2024_182_MOESM7_ESM.zip › 3A/3A section 2 left.tif]

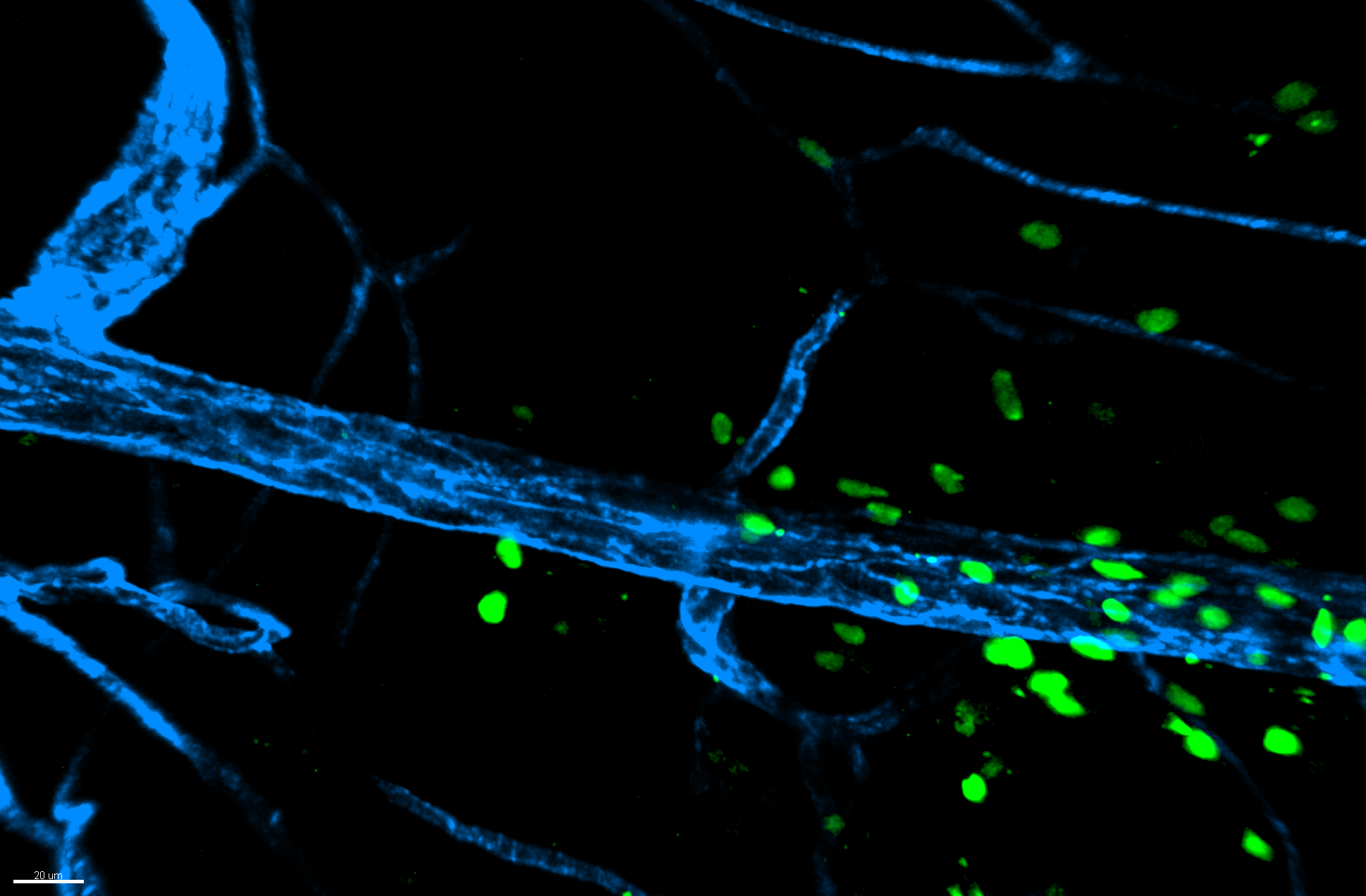

Supplement: Supplementary file 7 — Source data Fig. 3 [file 44319_2024_182_MOESM7_ESM.zip › 3A/3A section 2 right.tif]

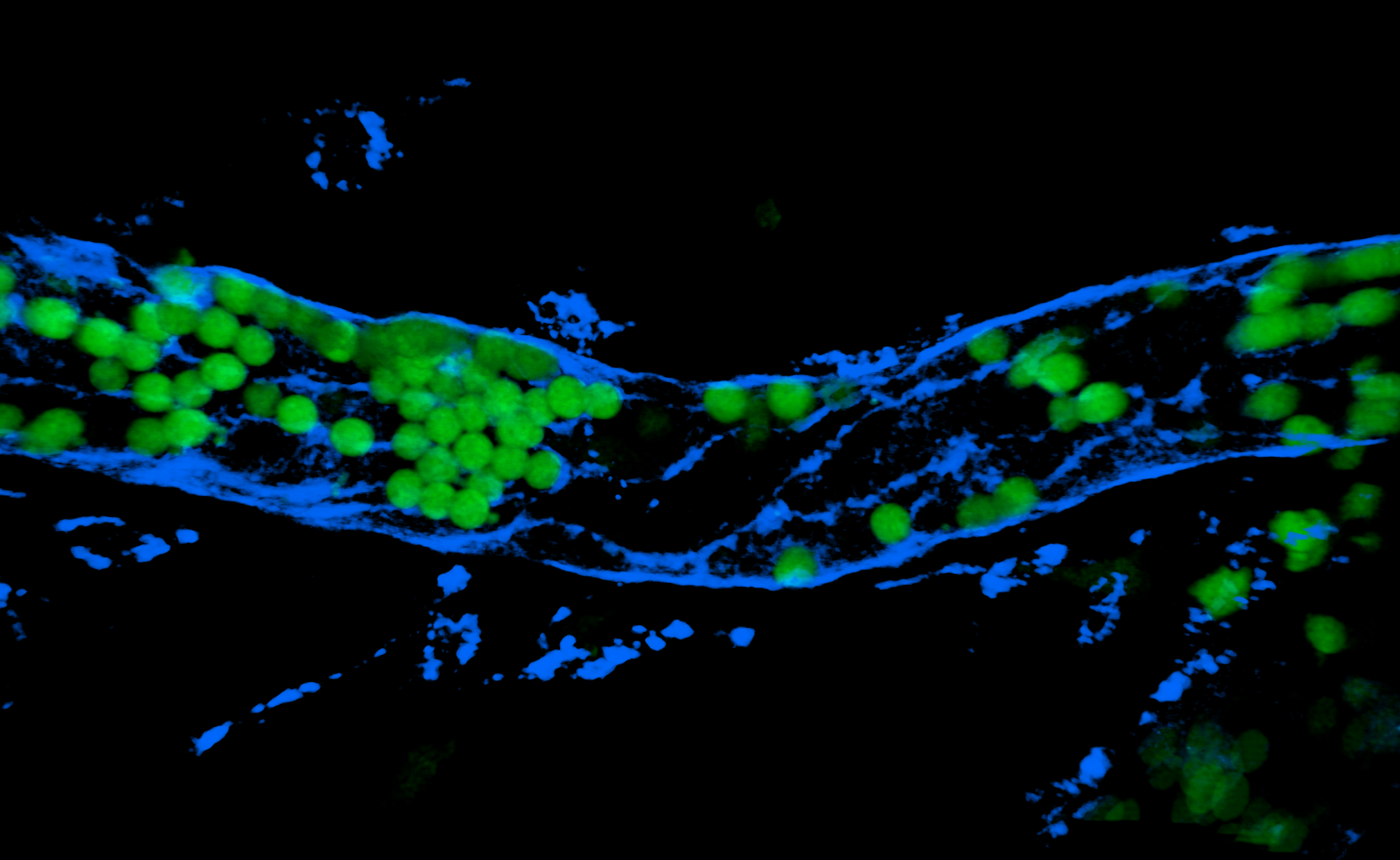

Supplement: Supplementary file 7 — Source data Fig. 3 [file 44319_2024_182_MOESM7_ESM.zip › 3C/3C bottom.tif]

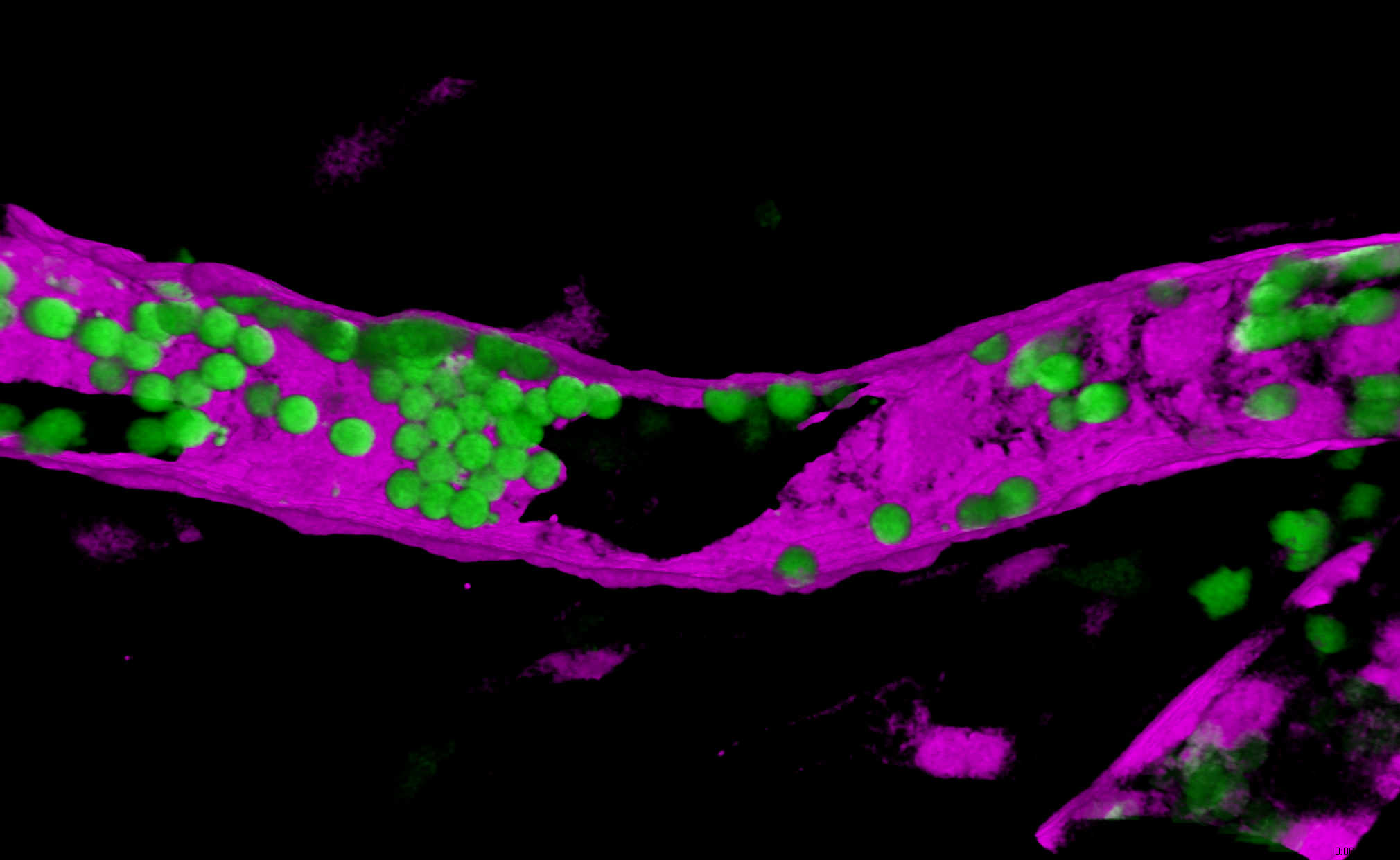

Supplement: Supplementary file 7 — Source data Fig. 3 [file 44319_2024_182_MOESM7_ESM.zip › 3C/3C top.tif]

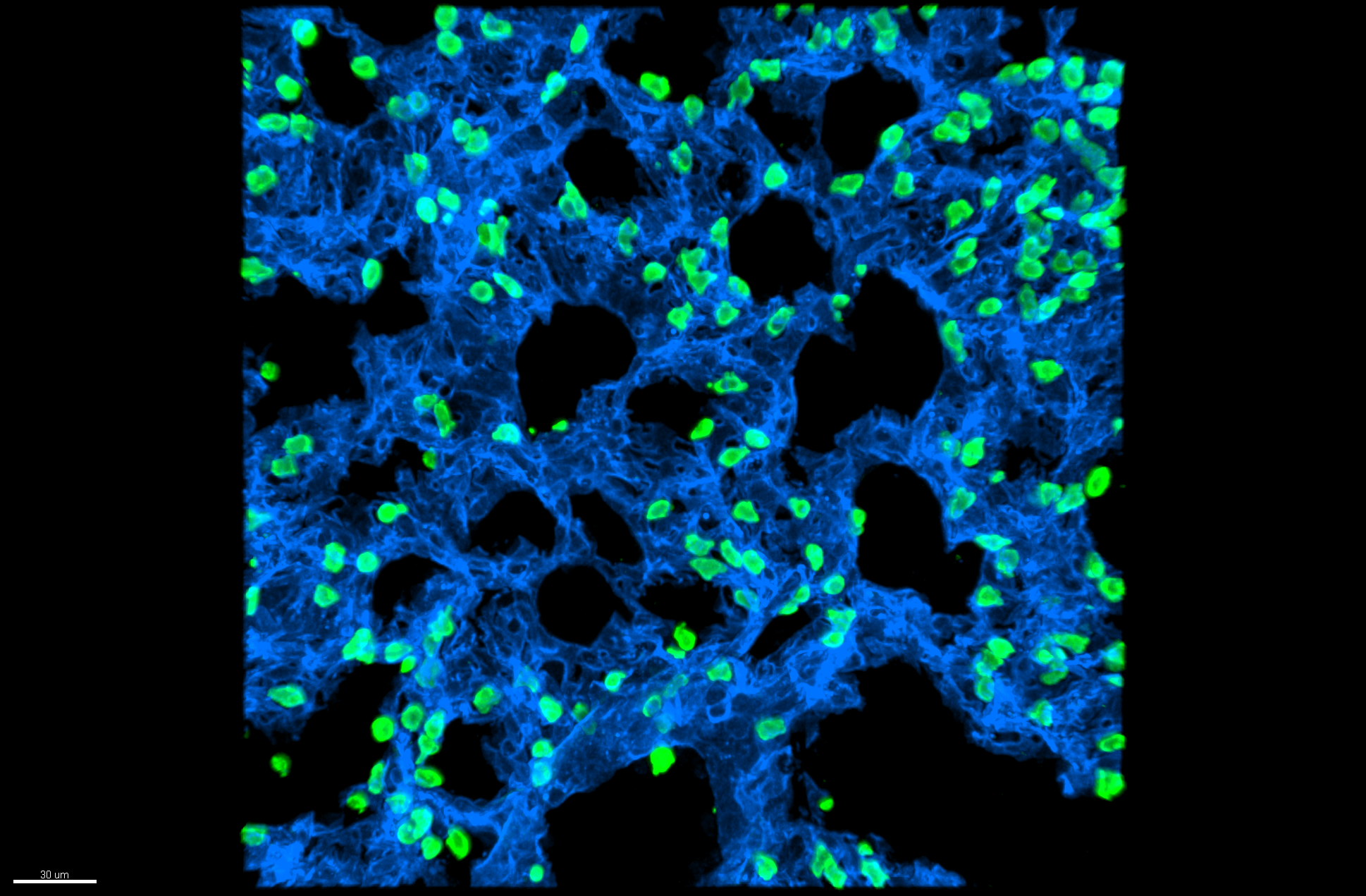

Supplement: Supplementary file 9 — Source data Fig. 5 [file 44319_2024_182_MOESM9_ESM.zip › 5C/Cre- LPS-PepG.tif]

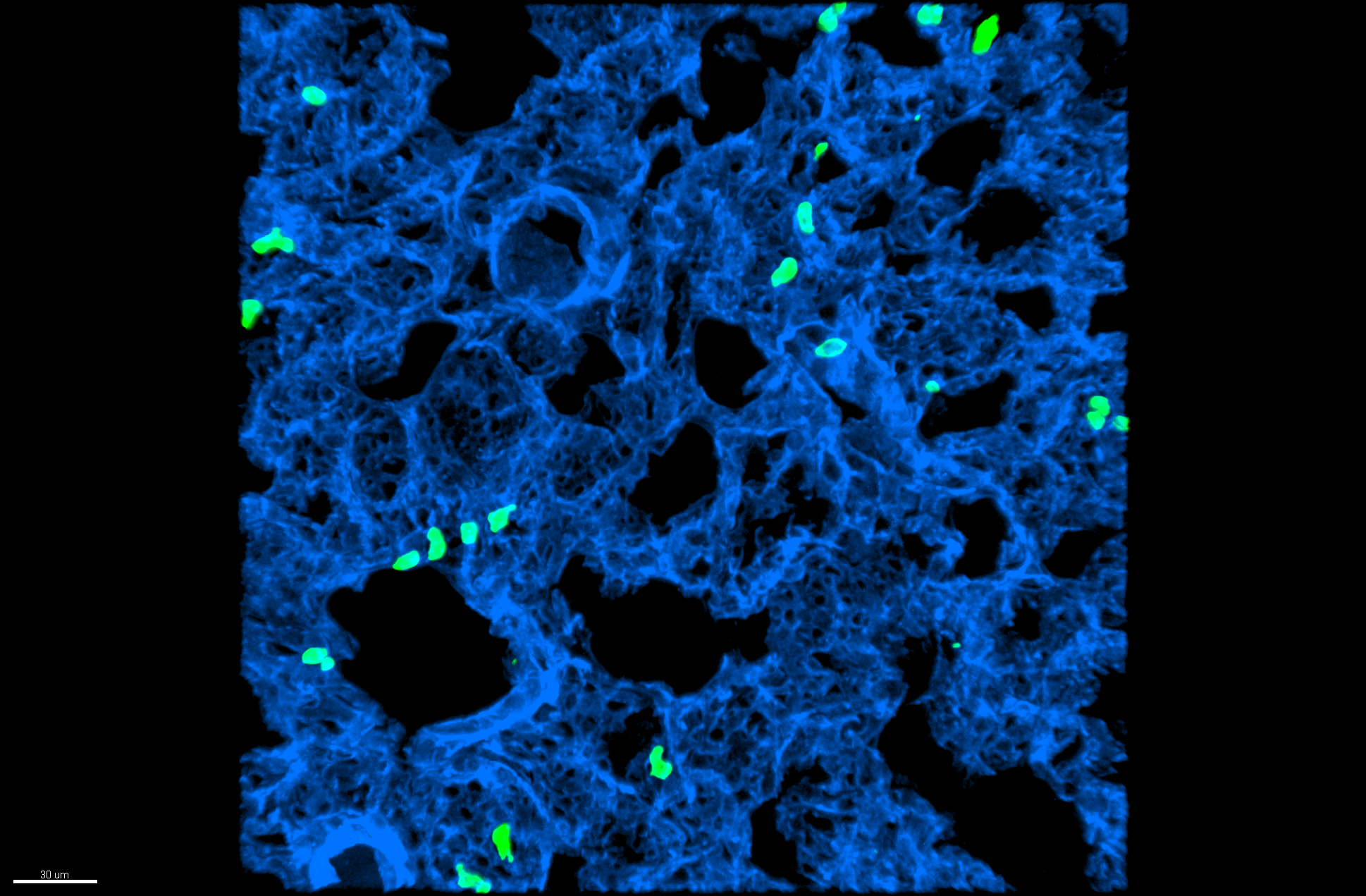

Supplement: Supplementary file 9 — Source data Fig. 5 [file 44319_2024_182_MOESM9_ESM.zip › 5C/Cre- PBS.tif]

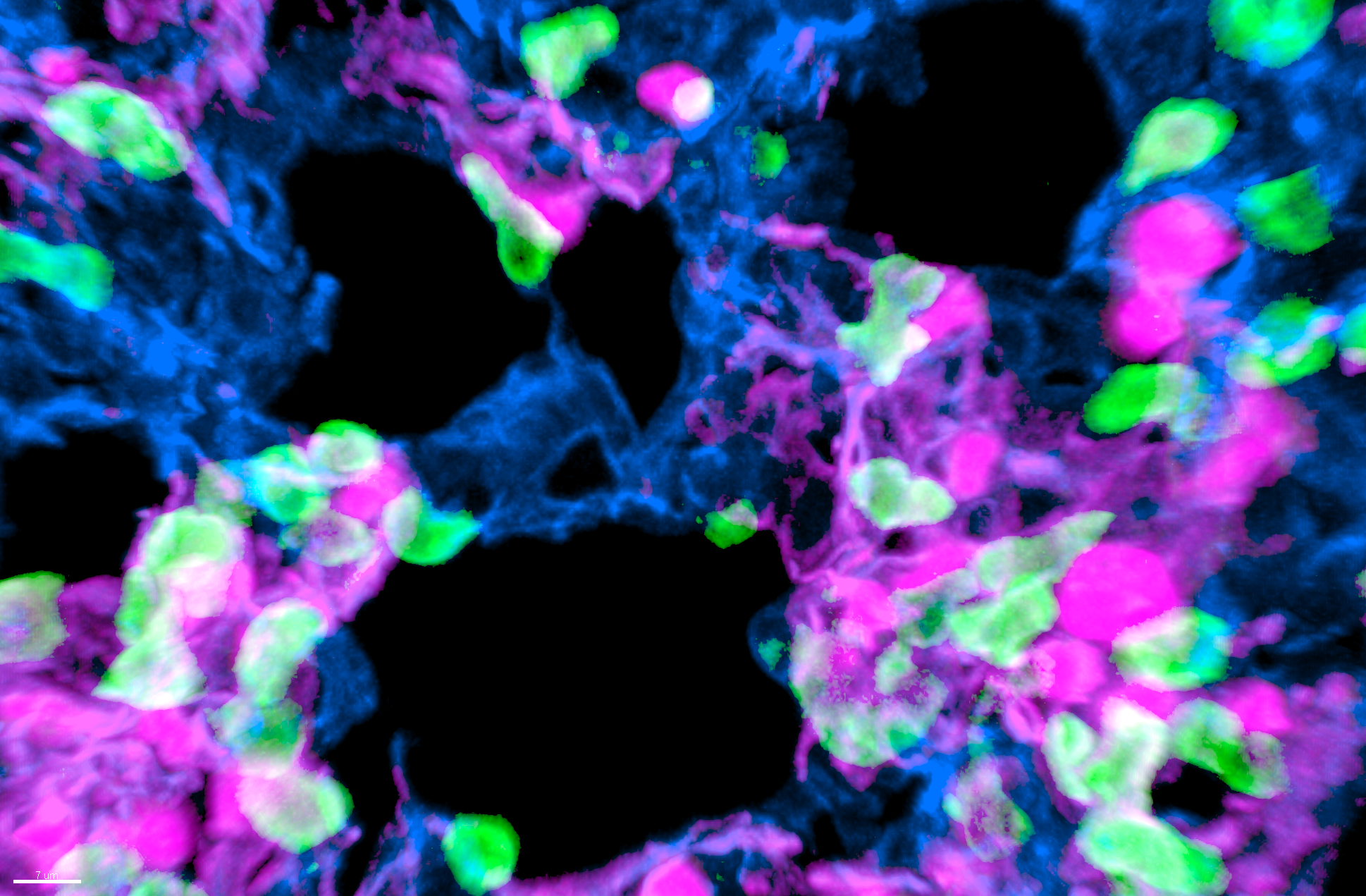

Supplement: Supplementary file 9 — Source data Fig. 5 [file 44319_2024_182_MOESM9_ESM.zip › 5C/Cre+ LPS-PepG high magnification 1.tif]

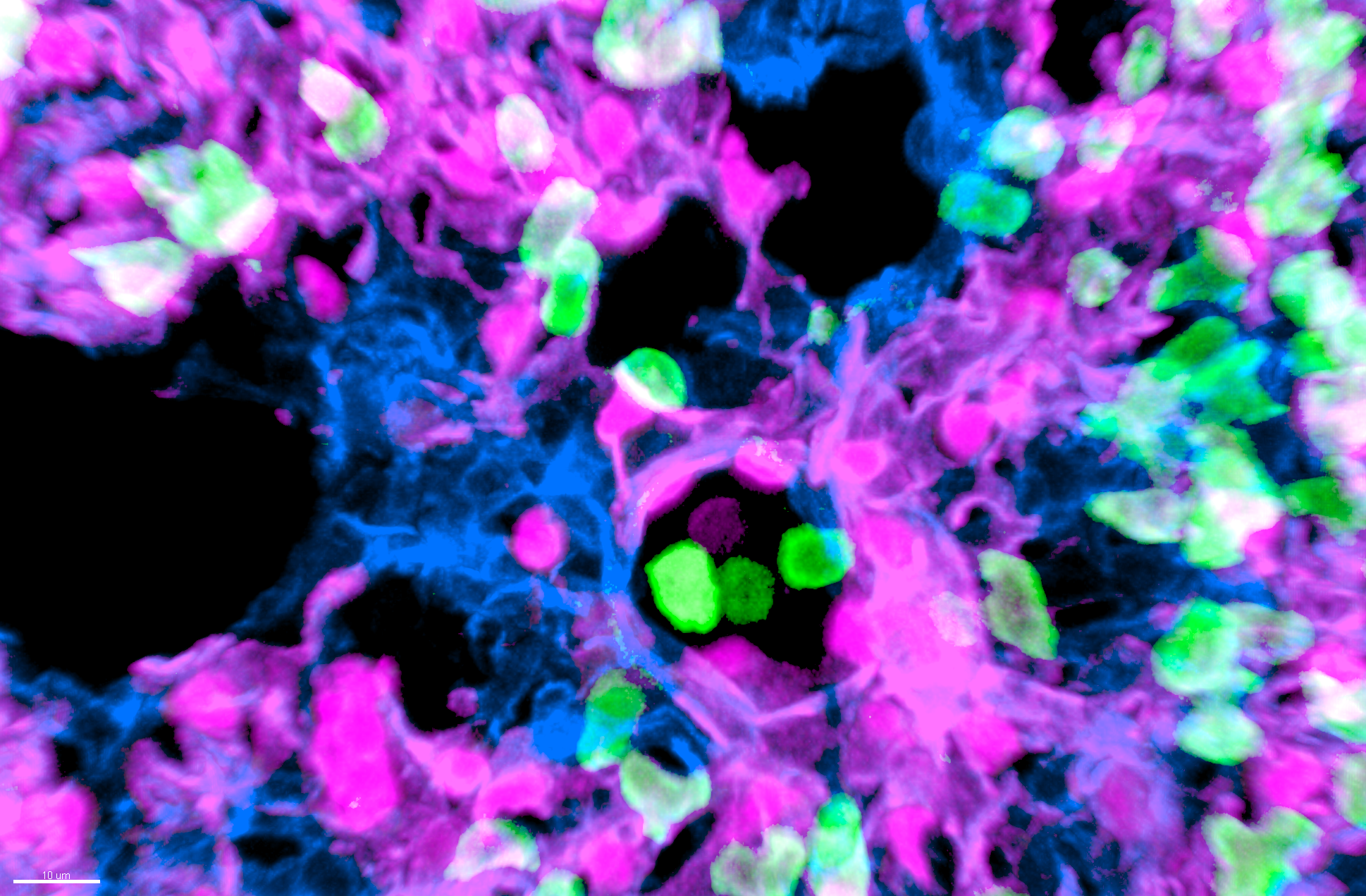

Supplement: Supplementary file 9 — Source data Fig. 5 [file 44319_2024_182_MOESM9_ESM.zip › 5C/Cre+ LPS-PepG high magnification 2.tif]

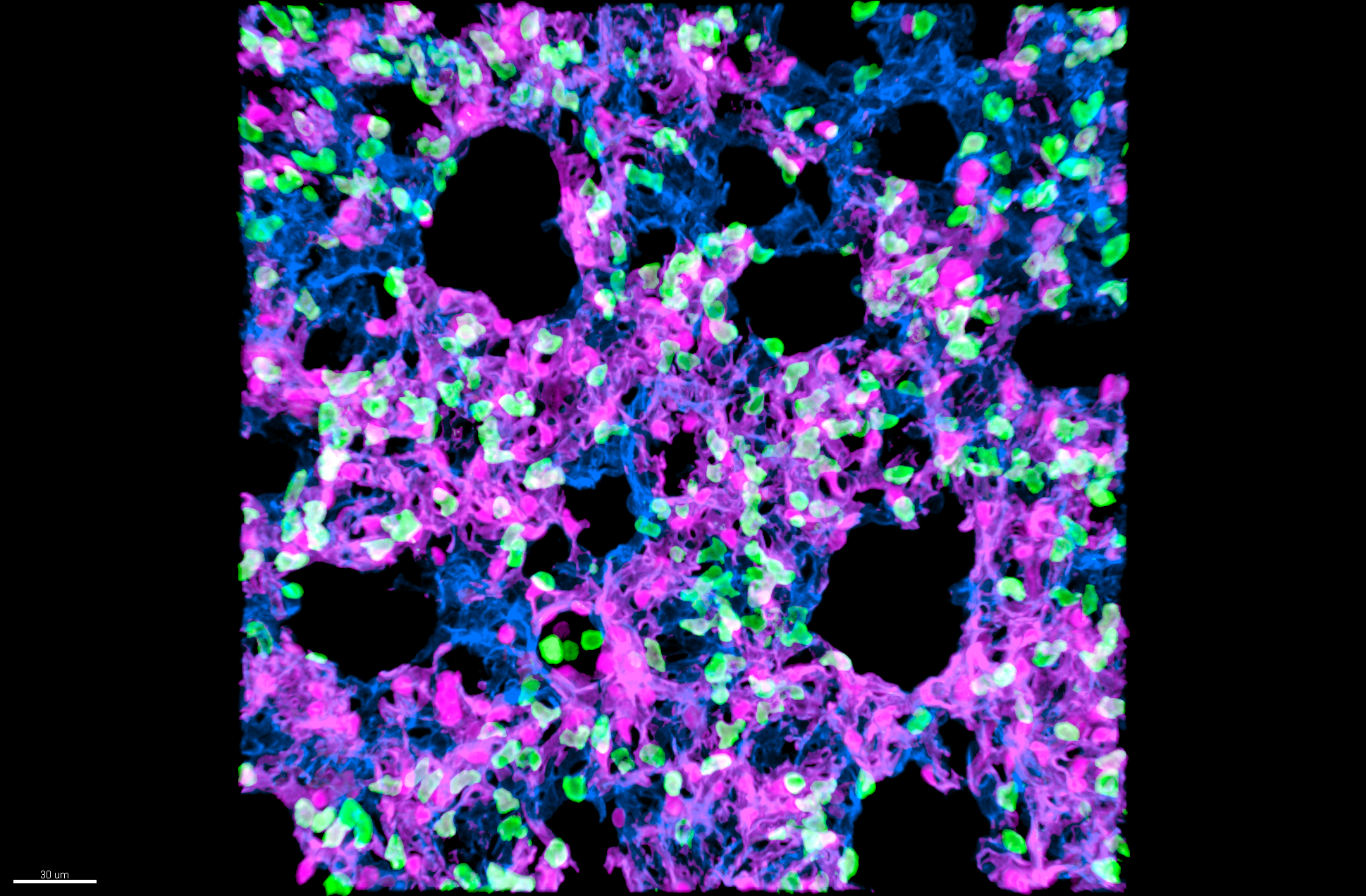

Supplement: Supplementary file 9 — Source data Fig. 5 [file 44319_2024_182_MOESM9_ESM.zip › 5C/Cre+ LPS-PepG.tif]

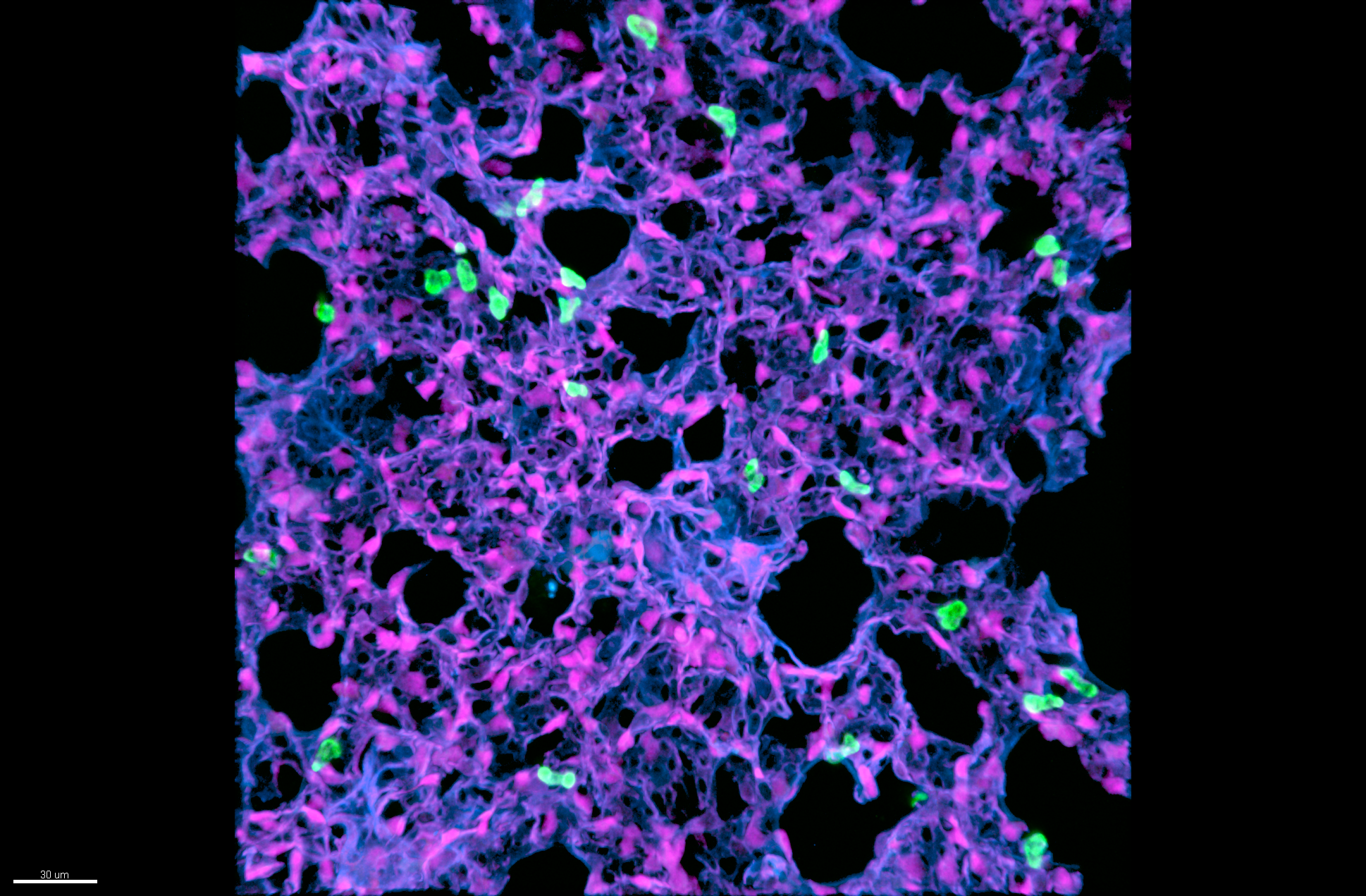

Supplement: Supplementary file 9 — Source data Fig. 5 [file 44319_2024_182_MOESM9_ESM.zip › 5C/Cre+ PBS.tif]

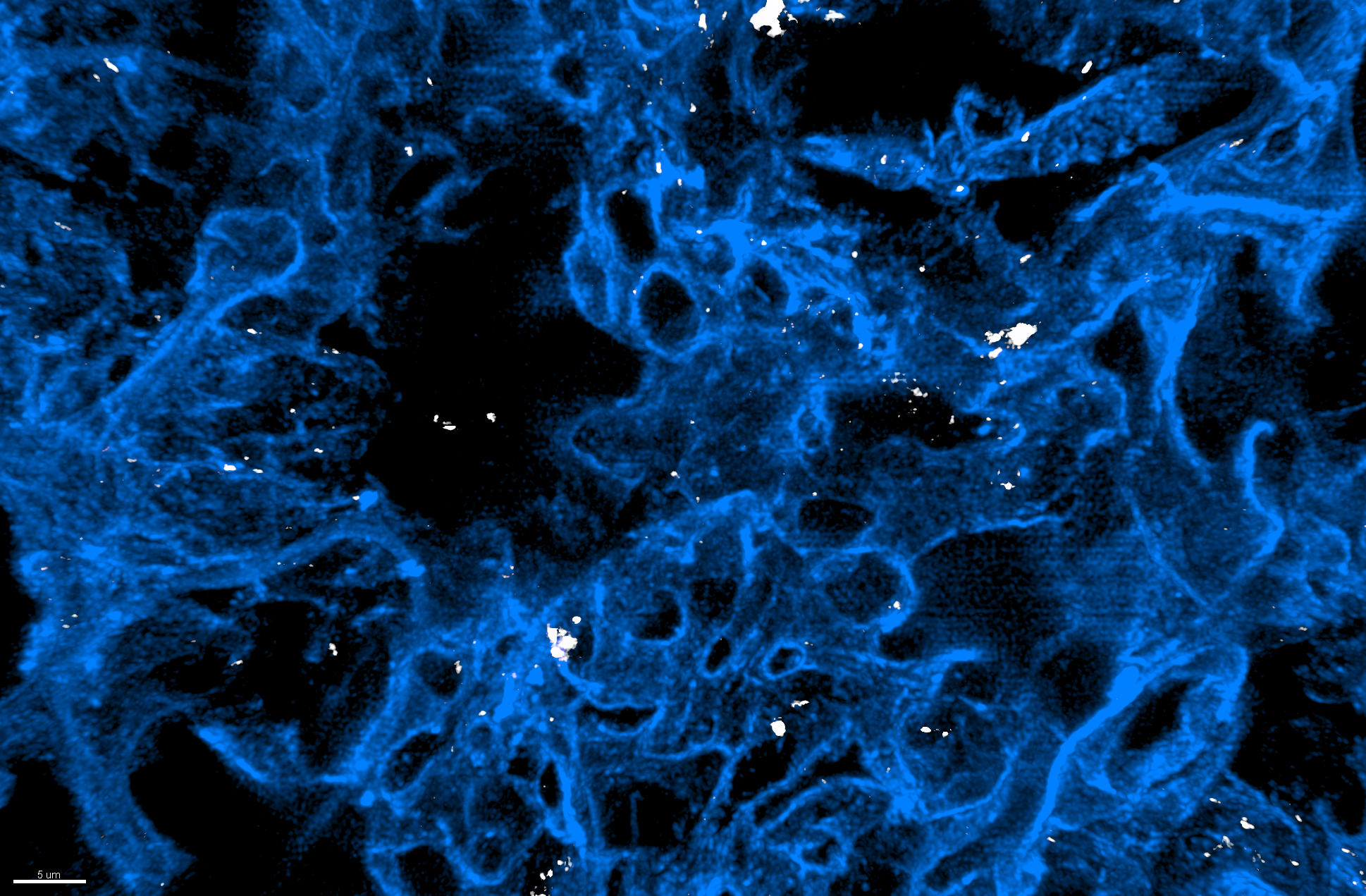

Supplement: Supplementary file 10 — Source data Fig. 6 [file 44319_2024_182_MOESM10_ESM.zip › 6D/Cre-.tif]

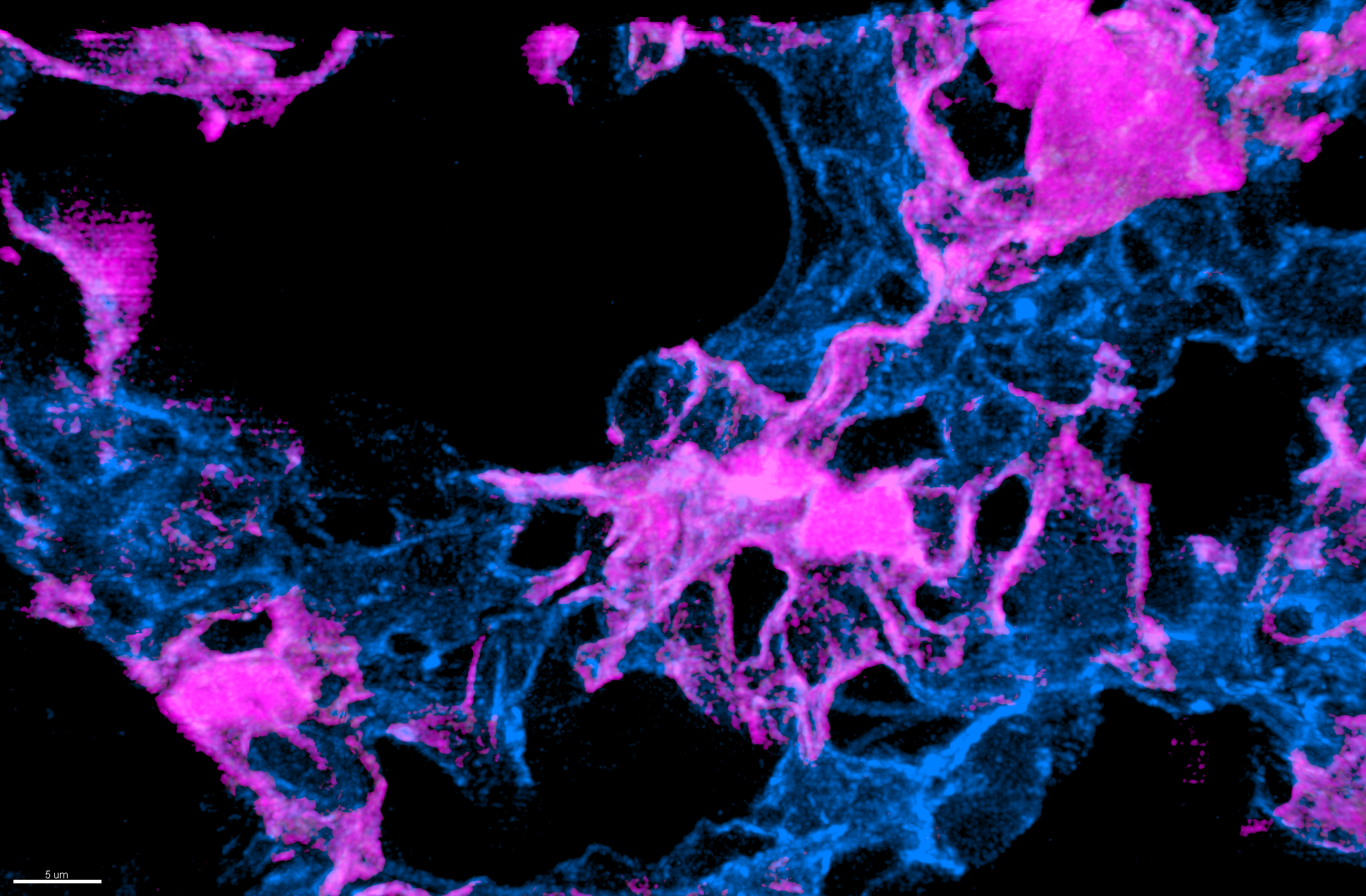

Supplement: Supplementary file 10 — Source data Fig. 6 [file 44319_2024_182_MOESM10_ESM.zip › 6D/Cre+ left.tif]

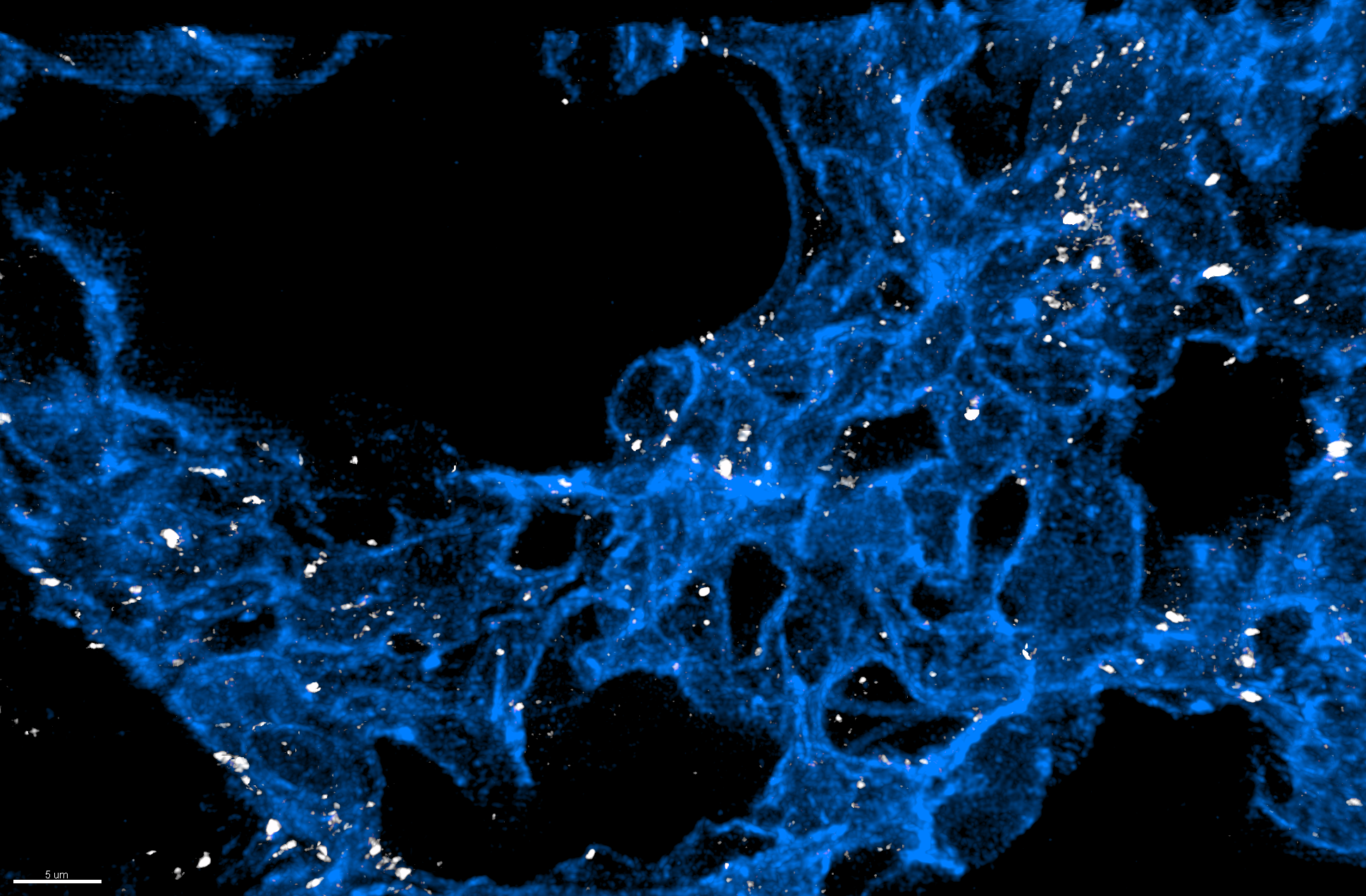

Supplement: Supplementary file 10 — Source data Fig. 6 [file 44319_2024_182_MOESM10_ESM.zip › 6D/Cre+ right.tif]

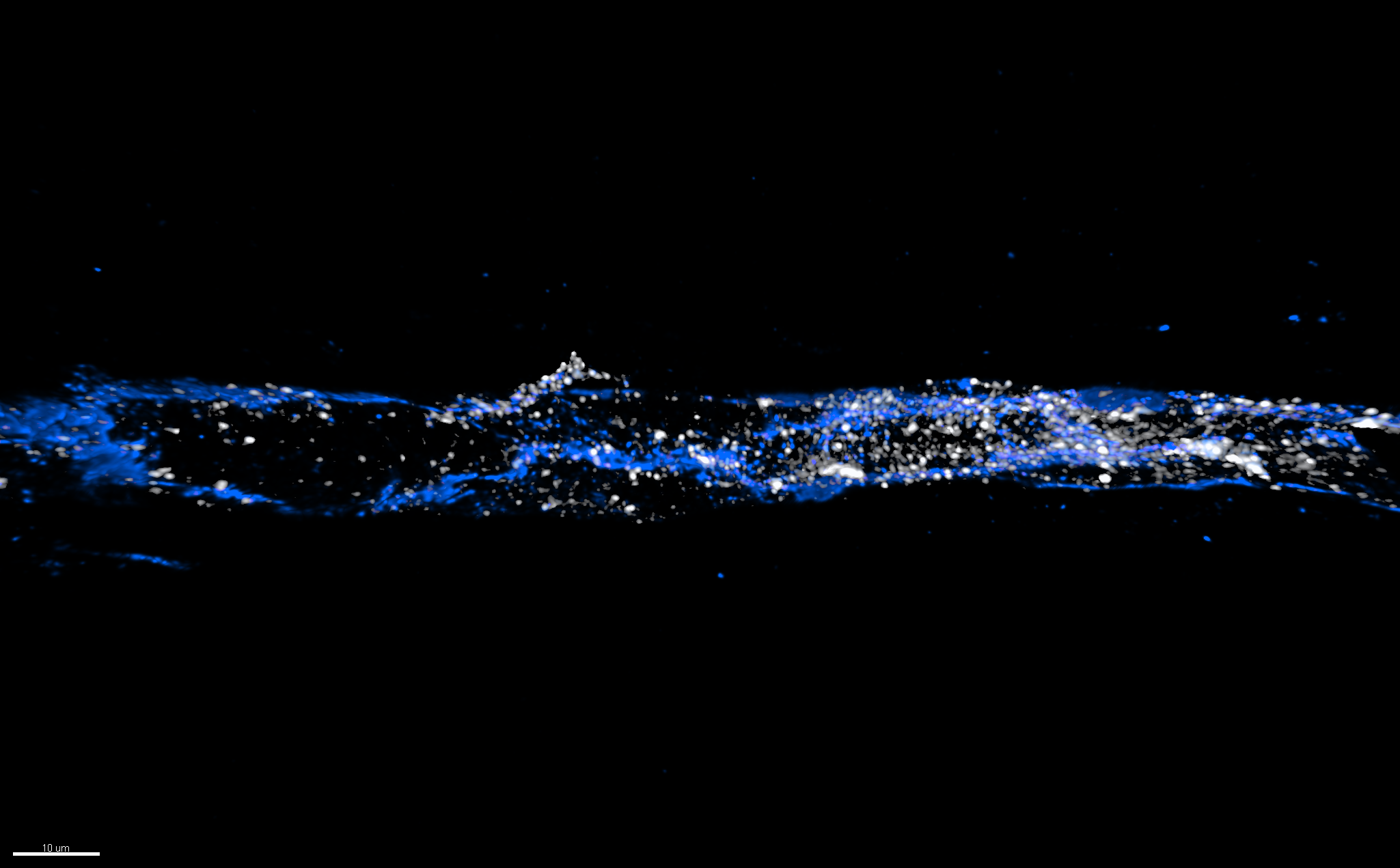

Supplement: Supplementary file 11 — Source data Fig. 7 [file 44319_2024_182_MOESM11_ESM.zip › 7E/7E bottom.tif]

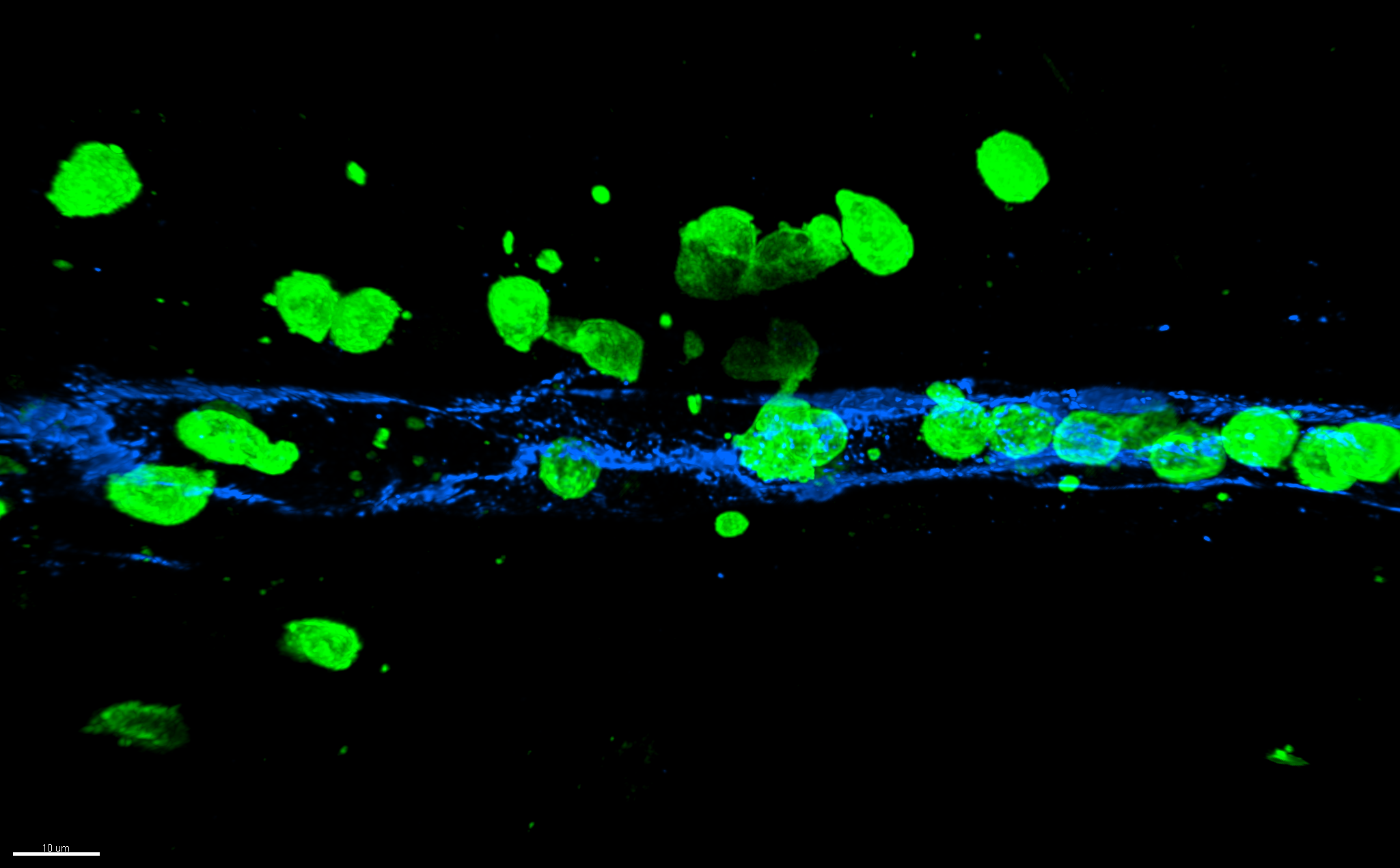

Supplement: Supplementary file 11 — Source data Fig. 7 [file 44319_2024_182_MOESM11_ESM.zip › 7E/7E middle.tif]

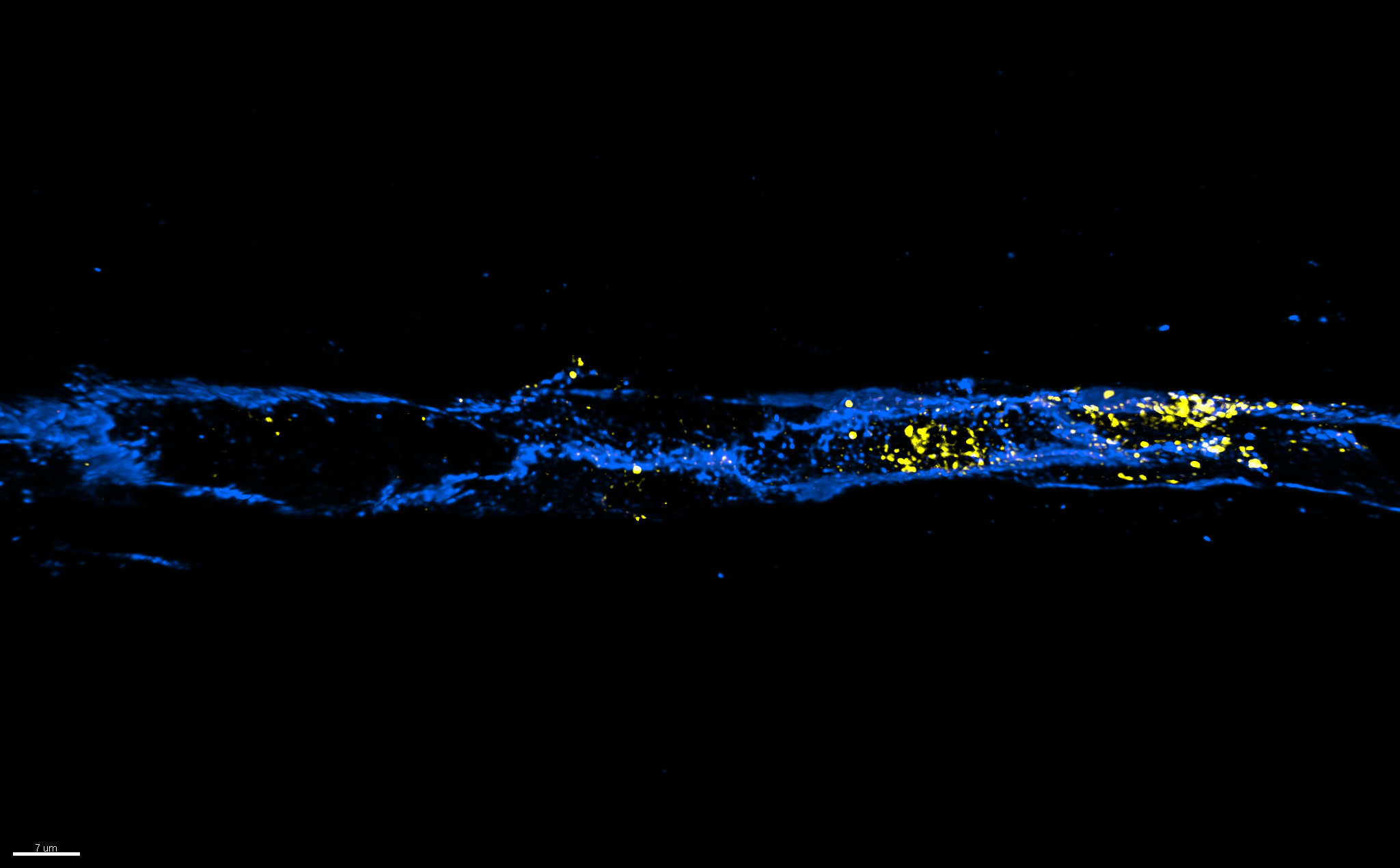

Supplement: Supplementary file 11 — Source data Fig. 7 [file 44319_2024_182_MOESM11_ESM.zip › 7E/7E top.tif]

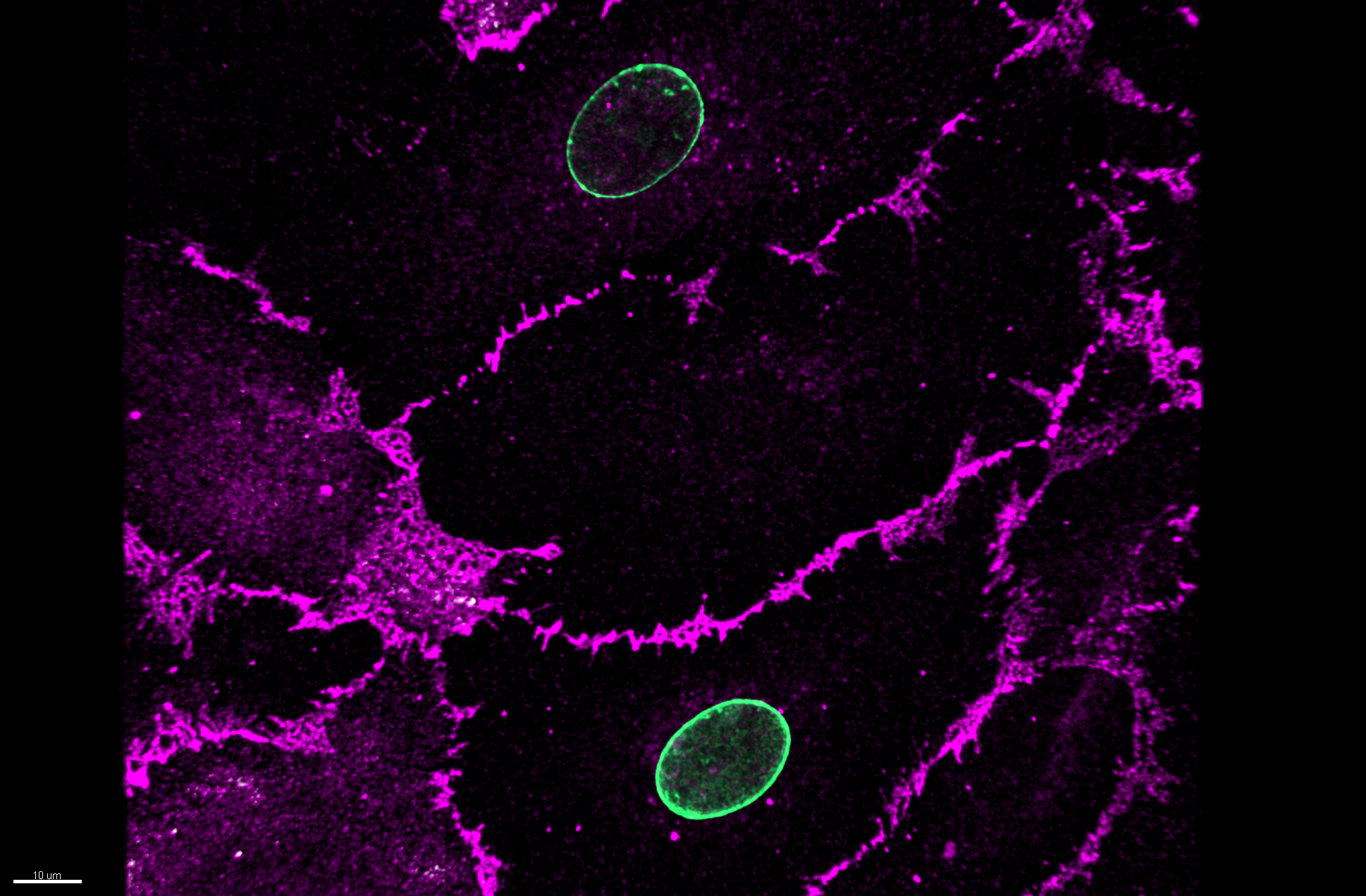

Supplement: Supplementary file 12 — Source data Fig. 8 [file 44319_2024_182_MOESM12_ESM.zip › 8A/Lamin A centre.tif]

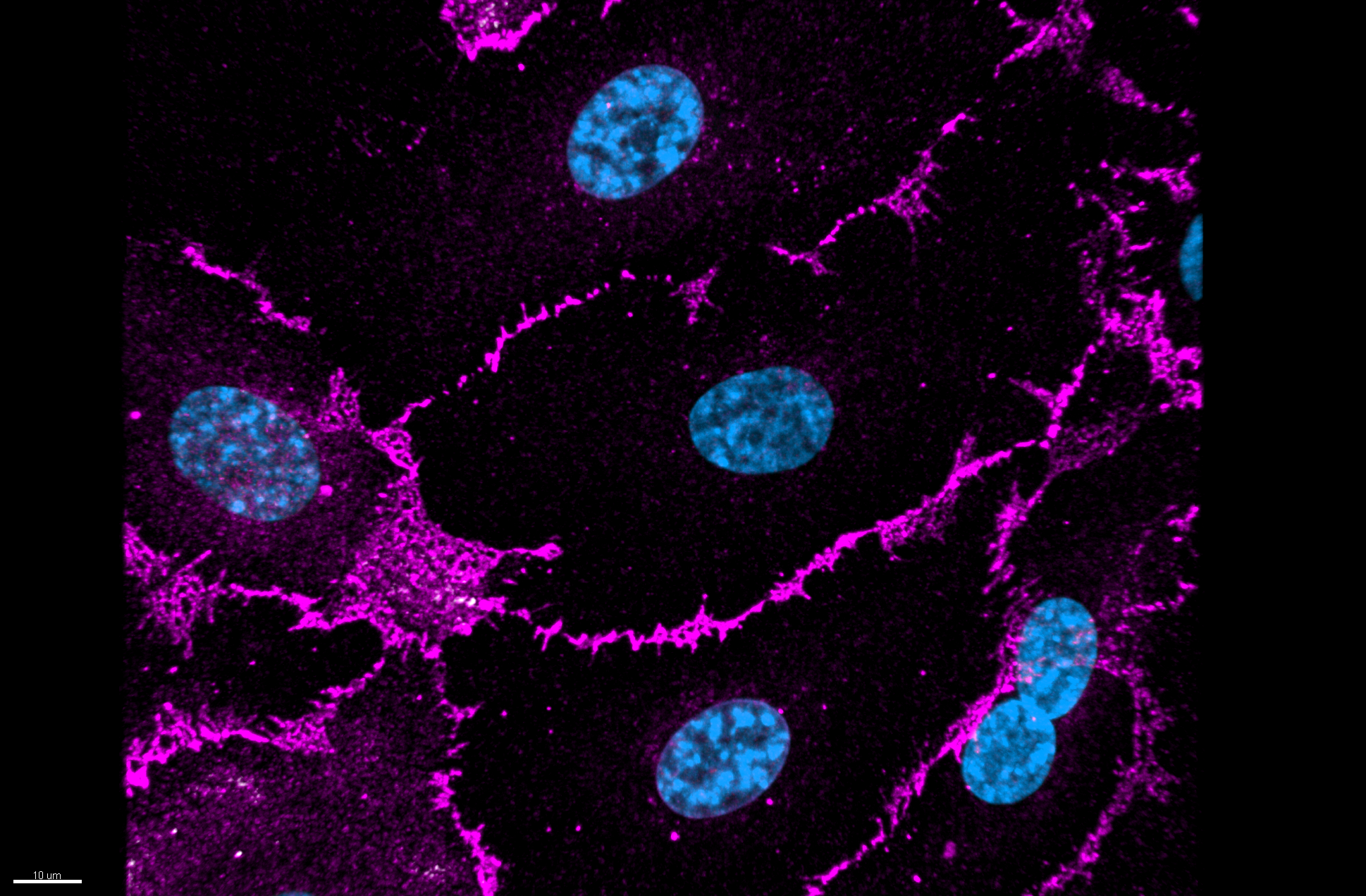

Supplement: Supplementary file 12 — Source data Fig. 8 [file 44319_2024_182_MOESM12_ESM.zip › 8A/Lamin A left.tif]

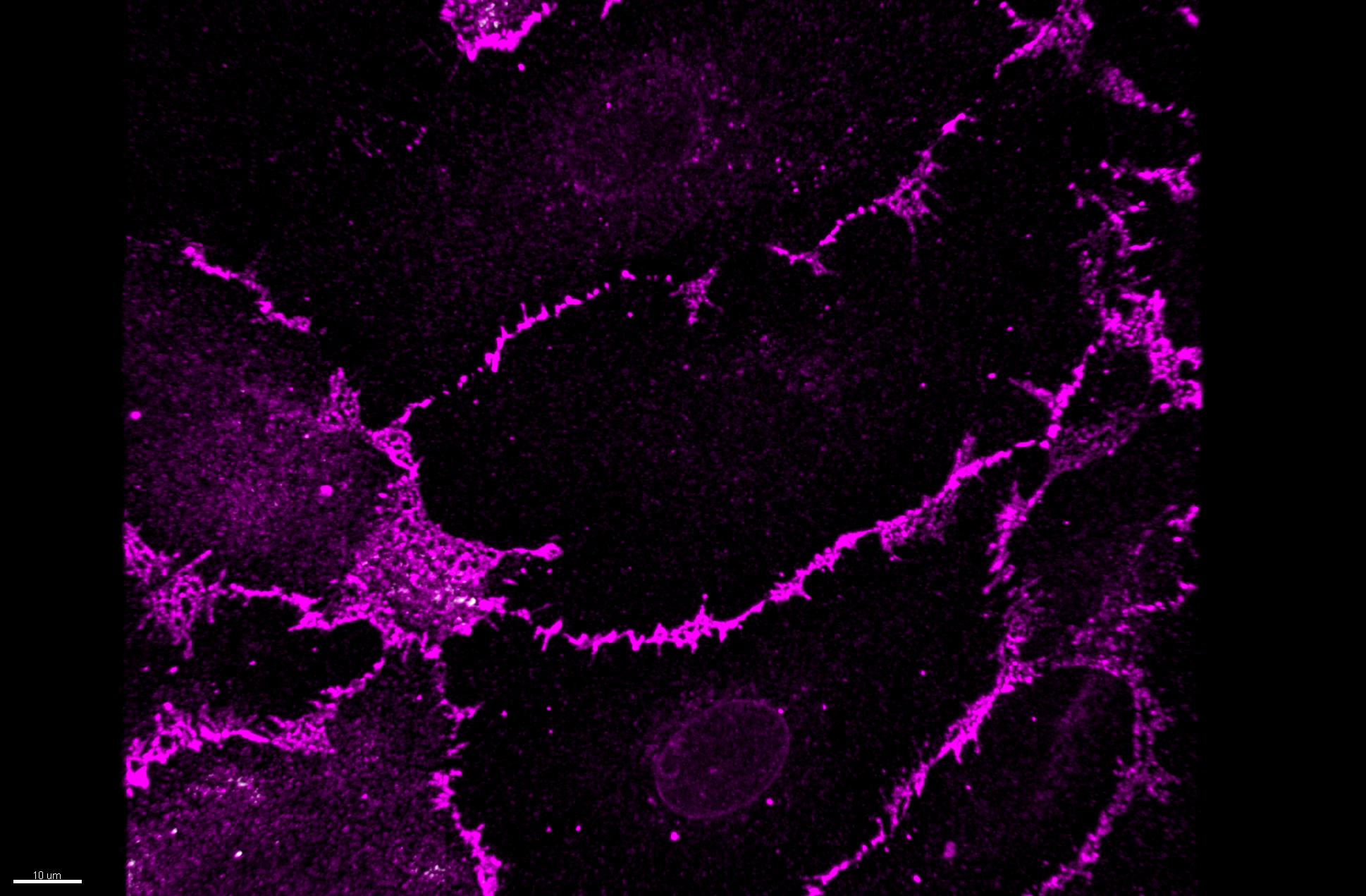

Supplement: Supplementary file 12 — Source data Fig. 8 [file 44319_2024_182_MOESM12_ESM.zip › 8A/Lamin A right.tif]

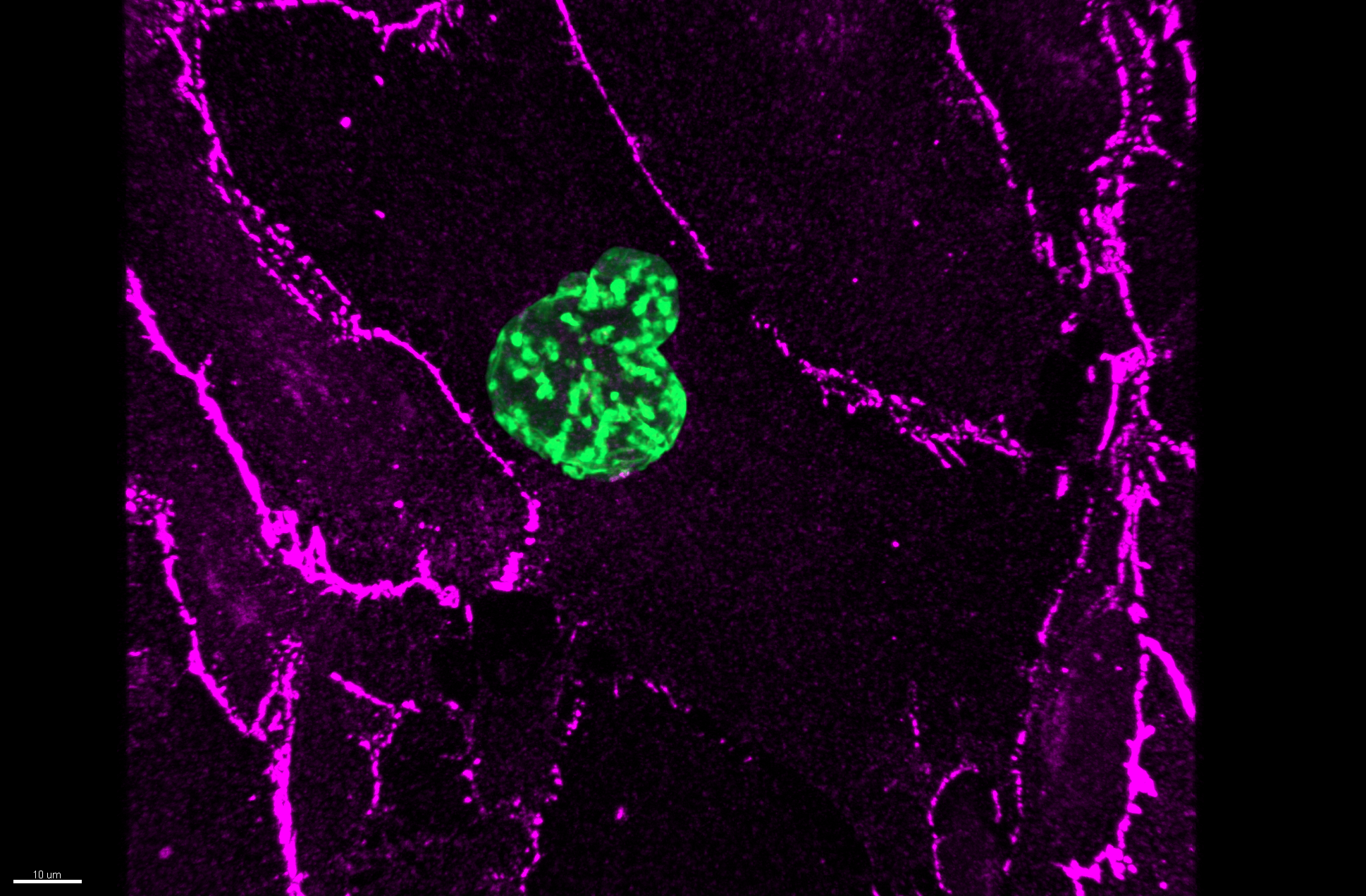

Supplement: Supplementary file 12 — Source data Fig. 8 [file 44319_2024_182_MOESM12_ESM.zip › 8A/Progerin centre.tif]

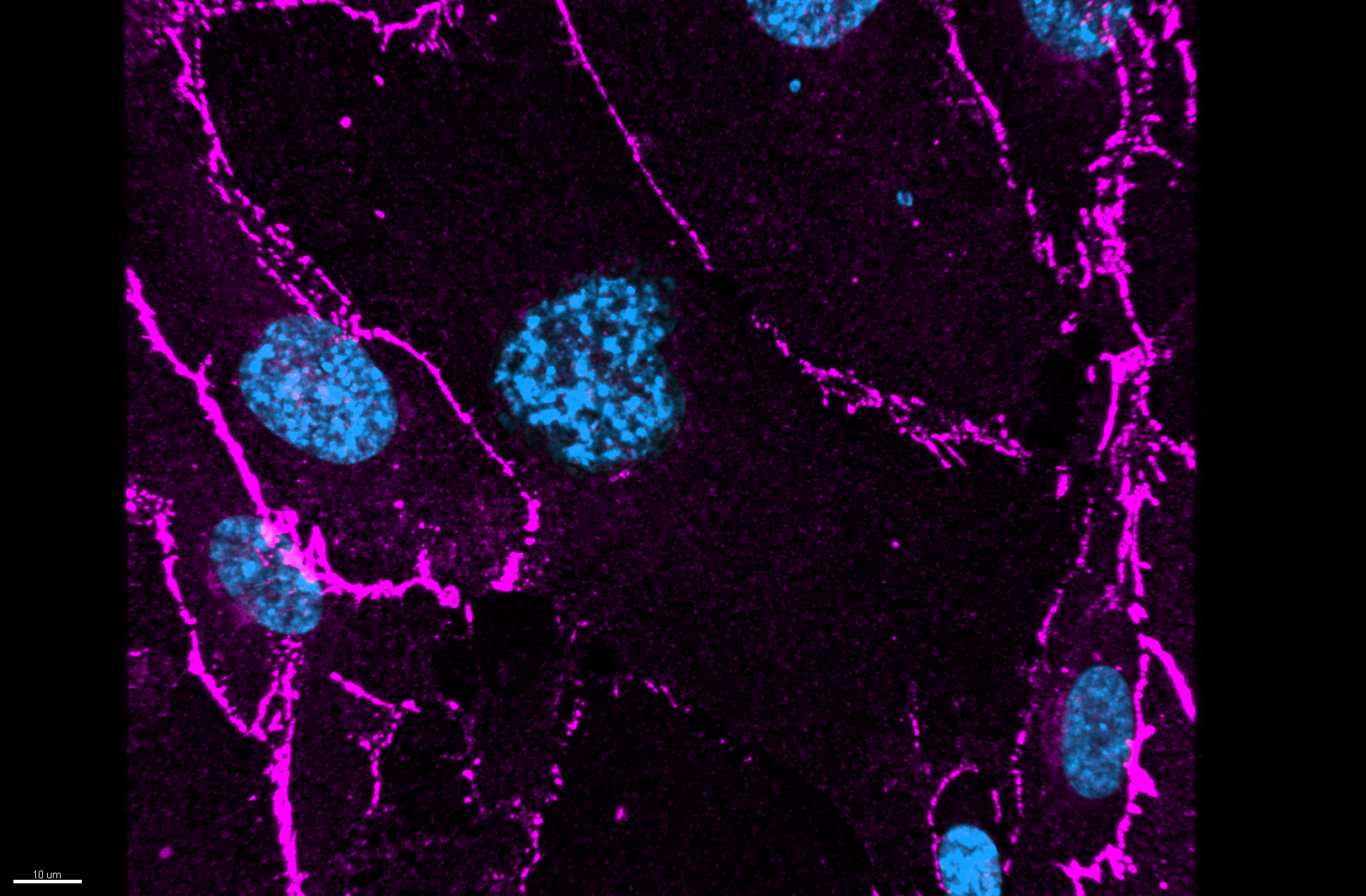

Supplement: Supplementary file 12 — Source data Fig. 8 [file 44319_2024_182_MOESM12_ESM.zip › 8A/Progerin left.tif]

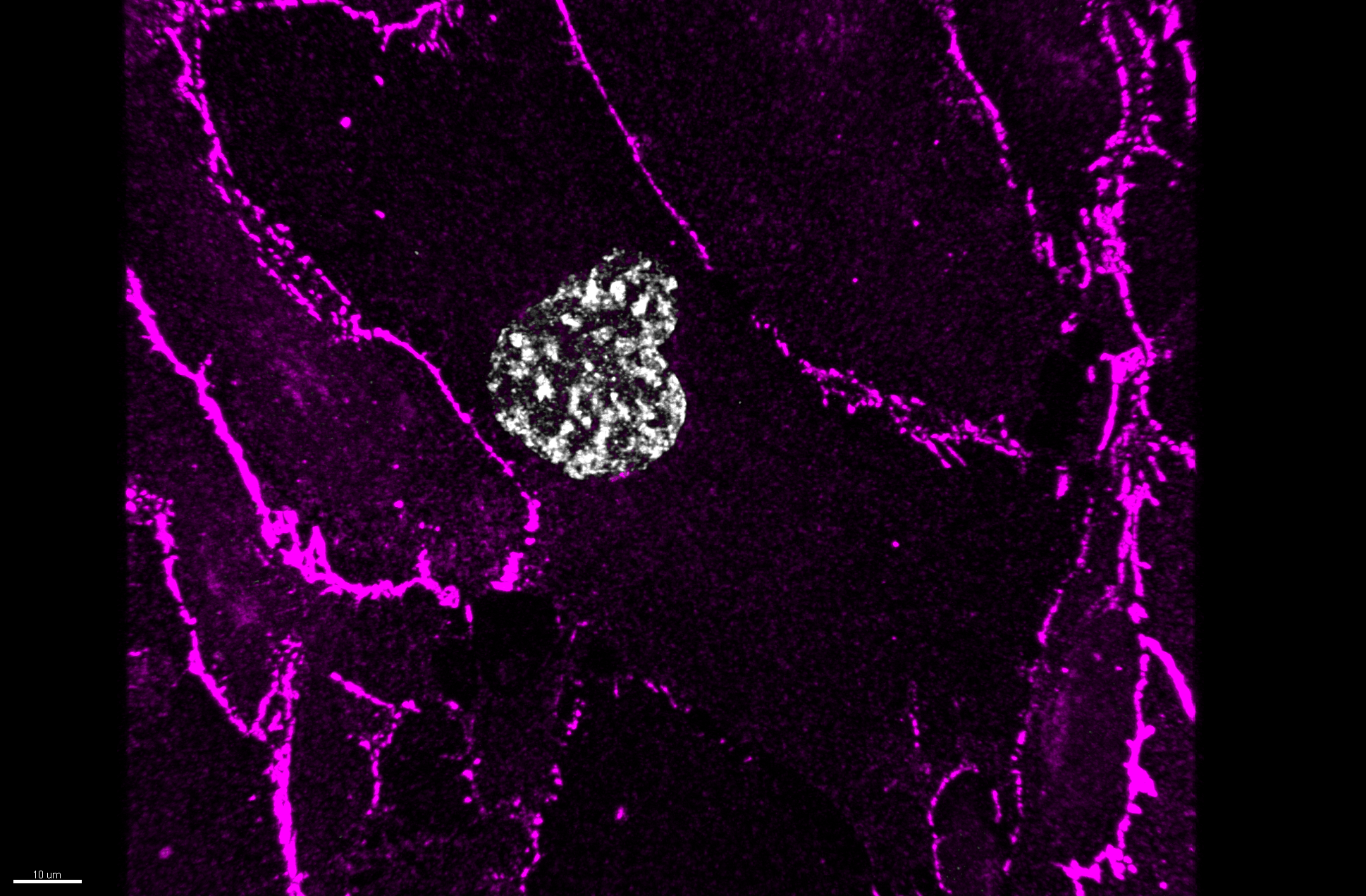

Supplement: Supplementary file 12 — Source data Fig. 8 [file 44319_2024_182_MOESM12_ESM.zip › 8A/Progerin right.tif]

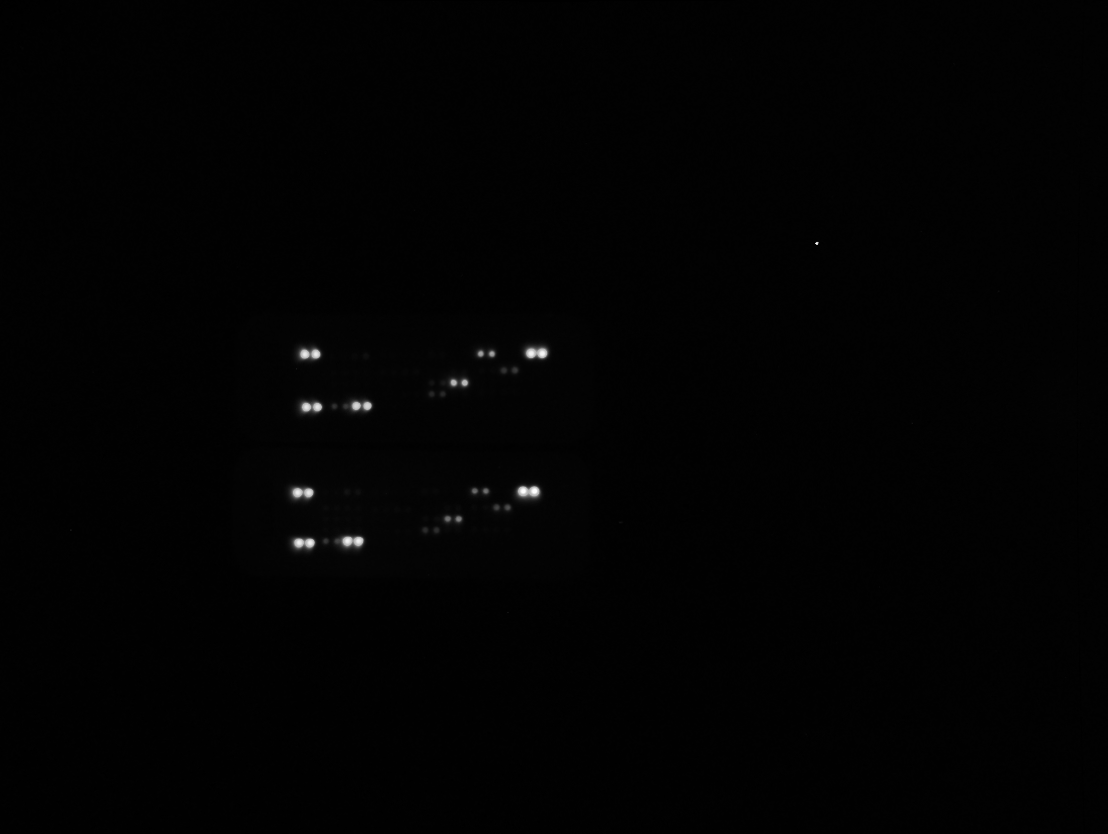

Supplement: Supplementary file 12 — Source data Fig. 8 [file 44319_2024_182_MOESM12_ESM.zip › 8C/IL-1b inverted.tif]

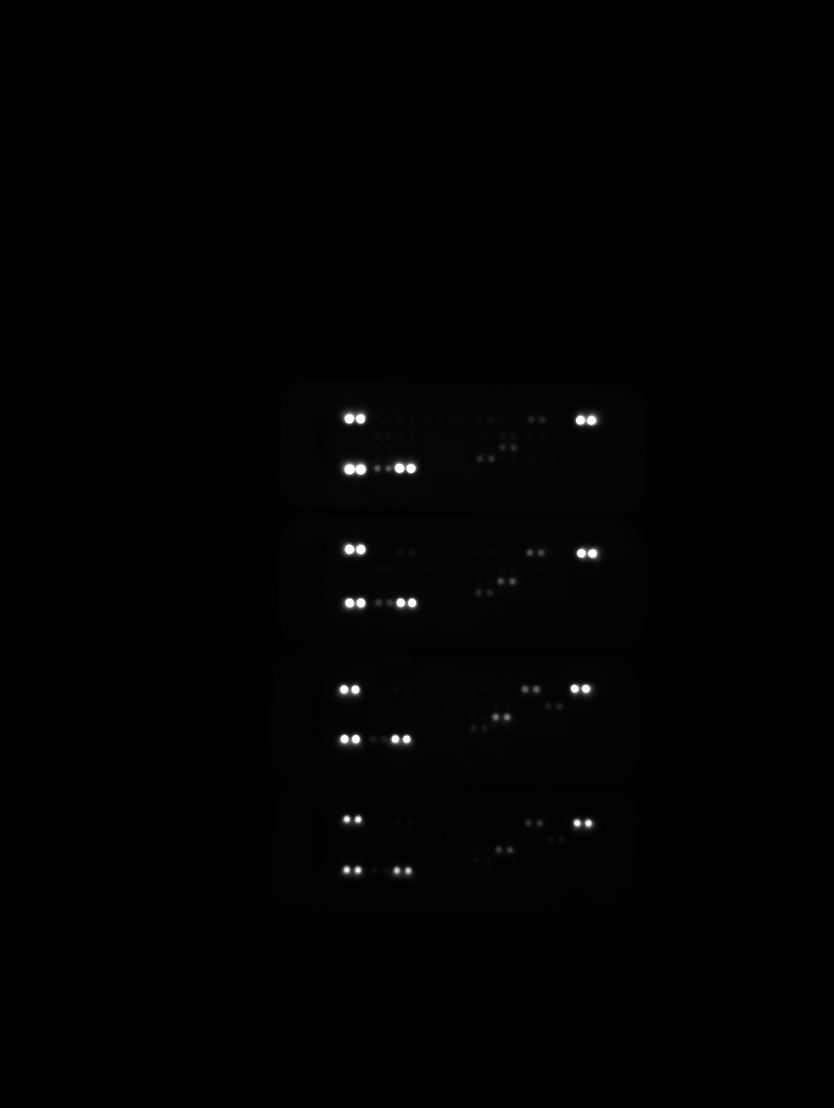

Supplement: Supplementary file 12 — Source data Fig. 8 [file 44319_2024_182_MOESM12_ESM.zip › 8C/PBS inverted.tif]

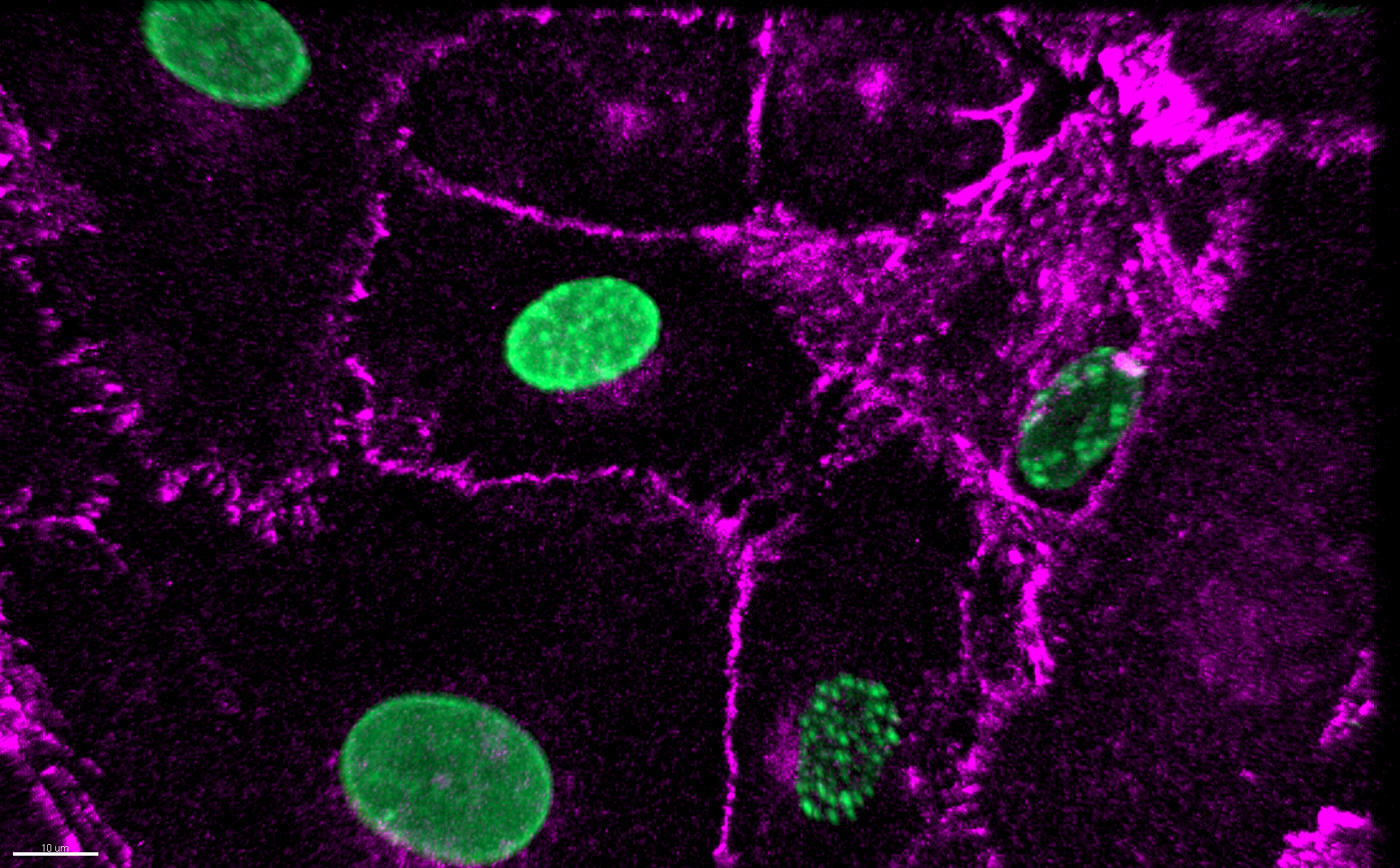

Supplement: Supplementary file 12 — Source data Fig. 8 [file 44319_2024_182_MOESM12_ESM.zip › 8E/Lamin A center.tif]

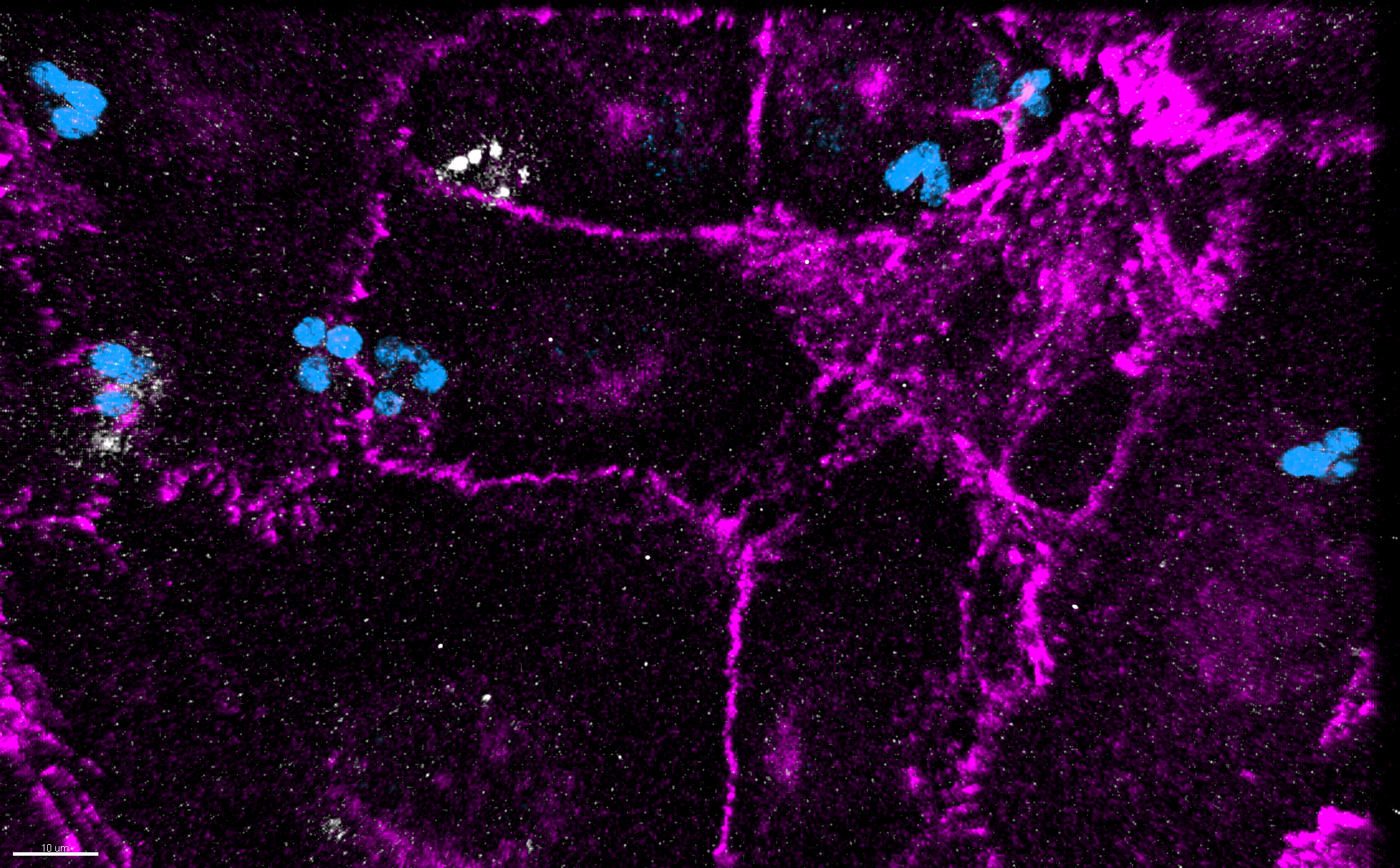

Supplement: Supplementary file 12 — Source data Fig. 8 [file 44319_2024_182_MOESM12_ESM.zip › 8E/Lamin A left.tif]

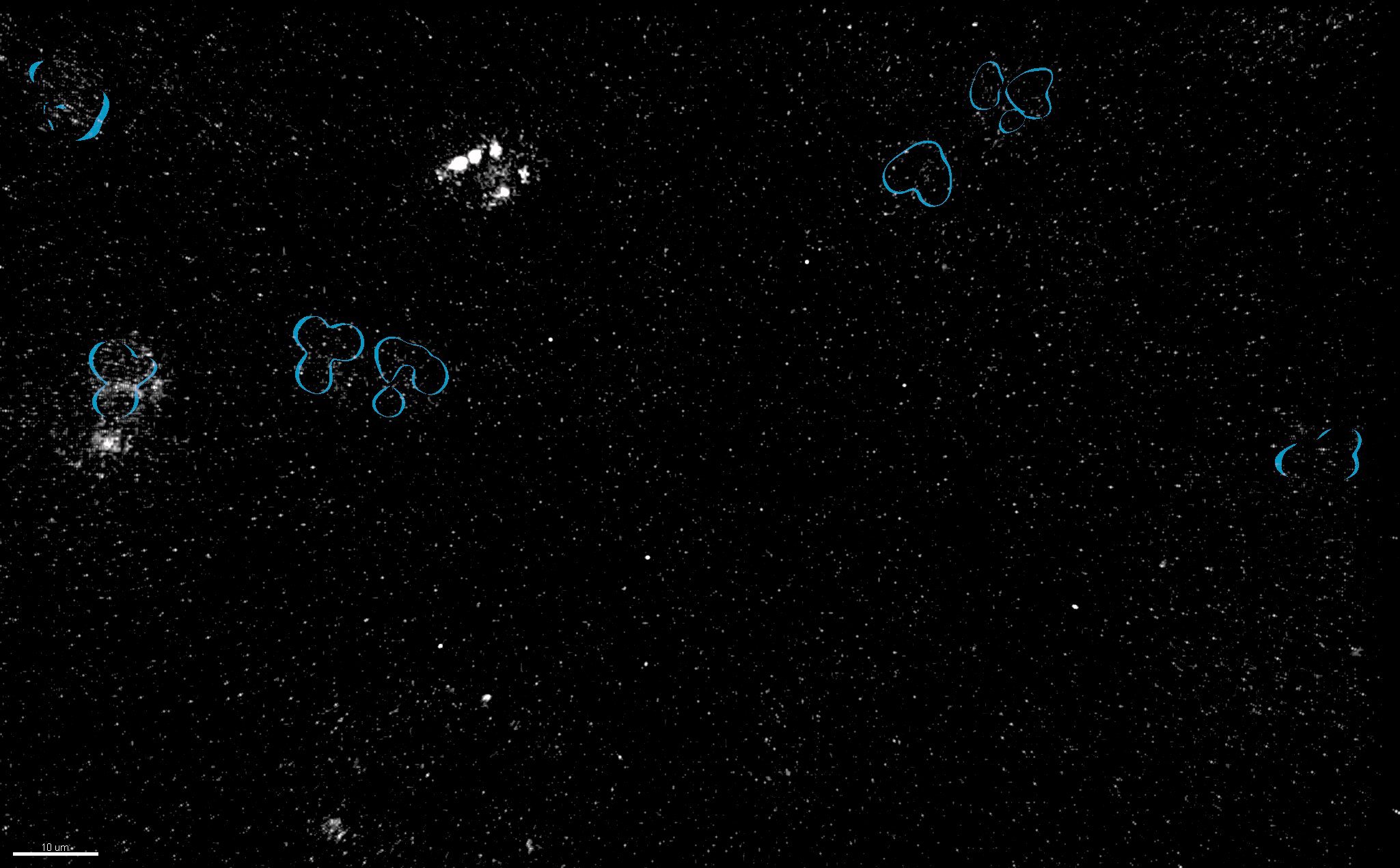

Supplement: Supplementary file 12 — Source data Fig. 8 [file 44319_2024_182_MOESM12_ESM.zip › 8E/Lamin A right.tif]

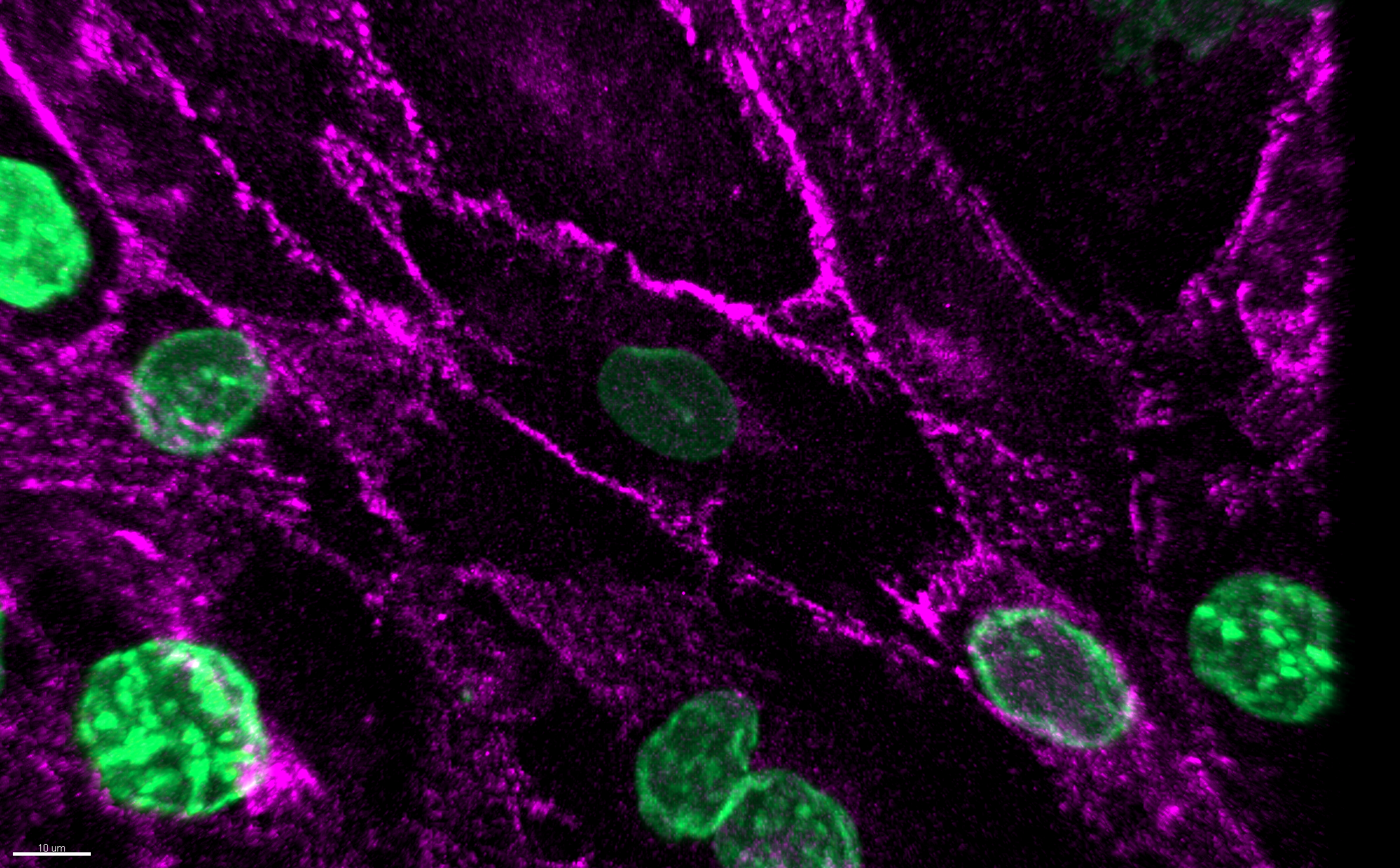

Supplement: Supplementary file 12 — Source data Fig. 8 [file 44319_2024_182_MOESM12_ESM.zip › 8E/Progerin centre.tif]

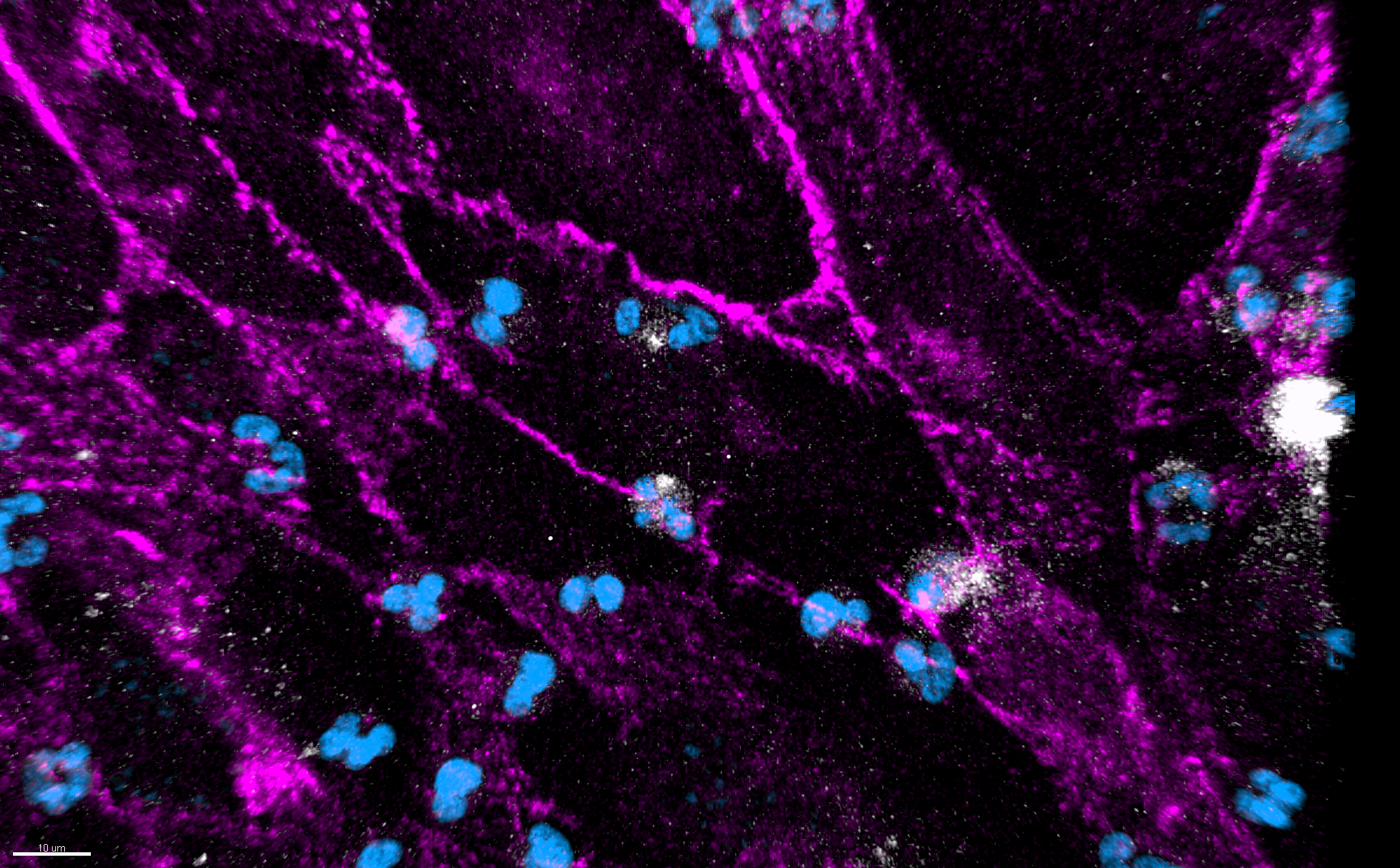

Supplement: Supplementary file 12 — Source data Fig. 8 [file 44319_2024_182_MOESM12_ESM.zip › 8E/Progerin left.tif]

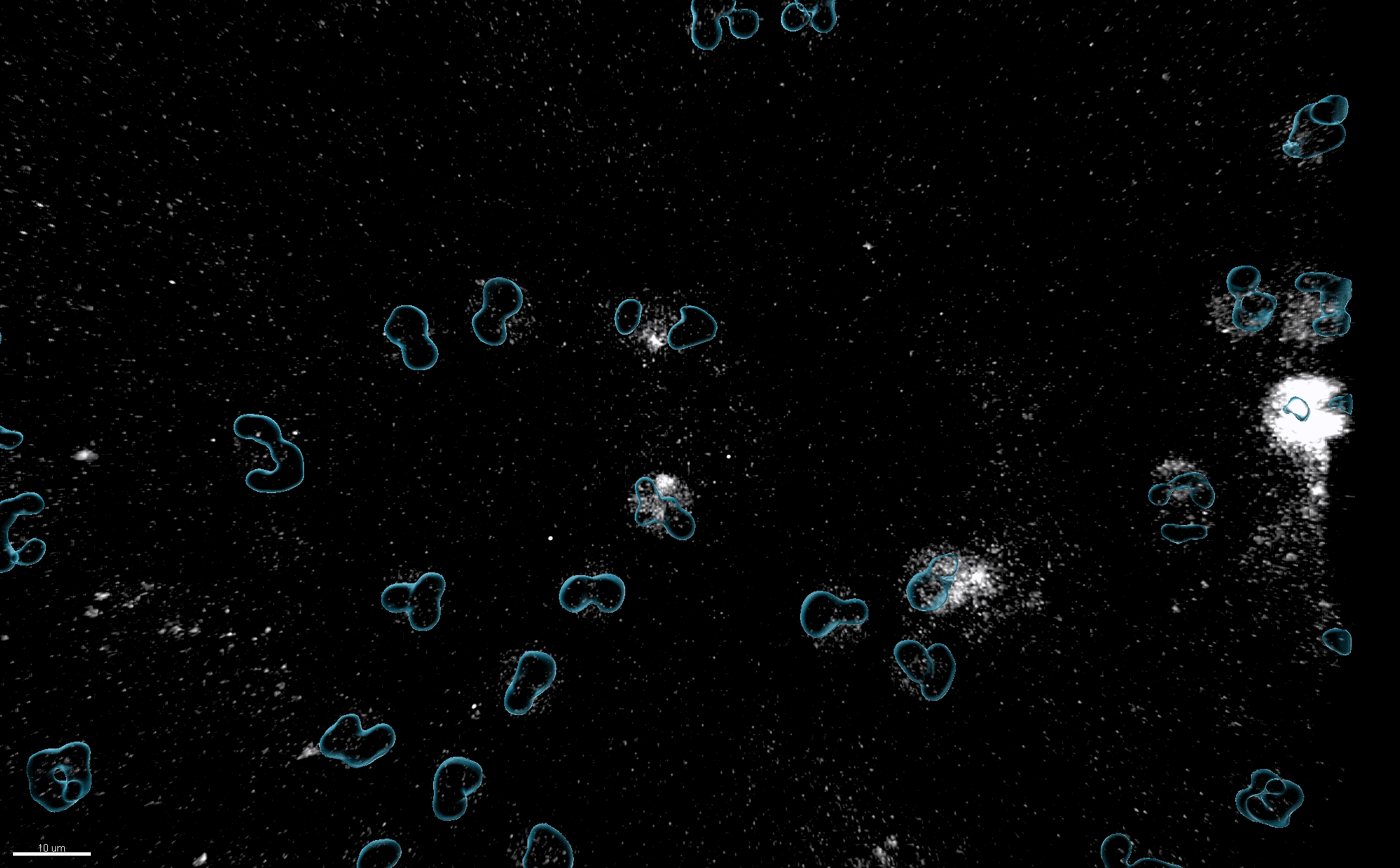

Supplement: Supplementary file 12 — Source data Fig. 8 [file 44319_2024_182_MOESM12_ESM.zip › 8E/Progerin right.tif]
