# Supplementary material for: Senescent endothelial cells promote pathogenic neutrophil trafficking in inflamed tissues
Source: EMBO Rep. 2024 Jun 25;25(9):10. doi: 10.1038/s44319-024-00182-x (PMC11387759; doi:10.1038/s44319-024-00182-x)
Supplement: Supplementary file 12 — Source data Fig. 8 [file 44319_2024_182_MOESM12_ESM.zip › 8C/Readme.docx]

Filename: PBS inverted.tif

- First from the top: Progerin-transduced HUVECS, PBS treated
- Second from the top: Lamin A-transduced HUVECS, PBS treated
- Third from the top: Was not used for the generation of the final figures
- First from the top: Was not used for the generation of the final figures

The last two membranes were defective, as evidenced by the lower intensity of the control spots. However, we were able to maintain the integrity of our experiment thanks to the manufacturer's prompt supply of new membranes to compensate for the loss of samples (filename = IL-1b inverted.tif).

Filename: IL-1b inverted.tif

- Top image = Progerin-transduced HUVECs
- Bottom image = Lamin A-transduced HUVECS
- Stimulation = IL-1β

The images shown are original captures from the camera. The colors were inverted using the FIJI software to generate the figures presented in the panel 8C
