# Supplementary material for: Senescent endothelial cells promote pathogenic neutrophil trafficking in inflamed tissues
Source: EMBO Rep. 2024 Jun 25;25(9):10. doi: 10.1038/s44319-024-00182-x (PMC11387759; doi:10.1038/s44319-024-00182-x)
Supplement: Supplementary file 13 — Expanded View Figures [file 44319_2024_182_MOESM13_ESM.pdf]

Expanded View Figures

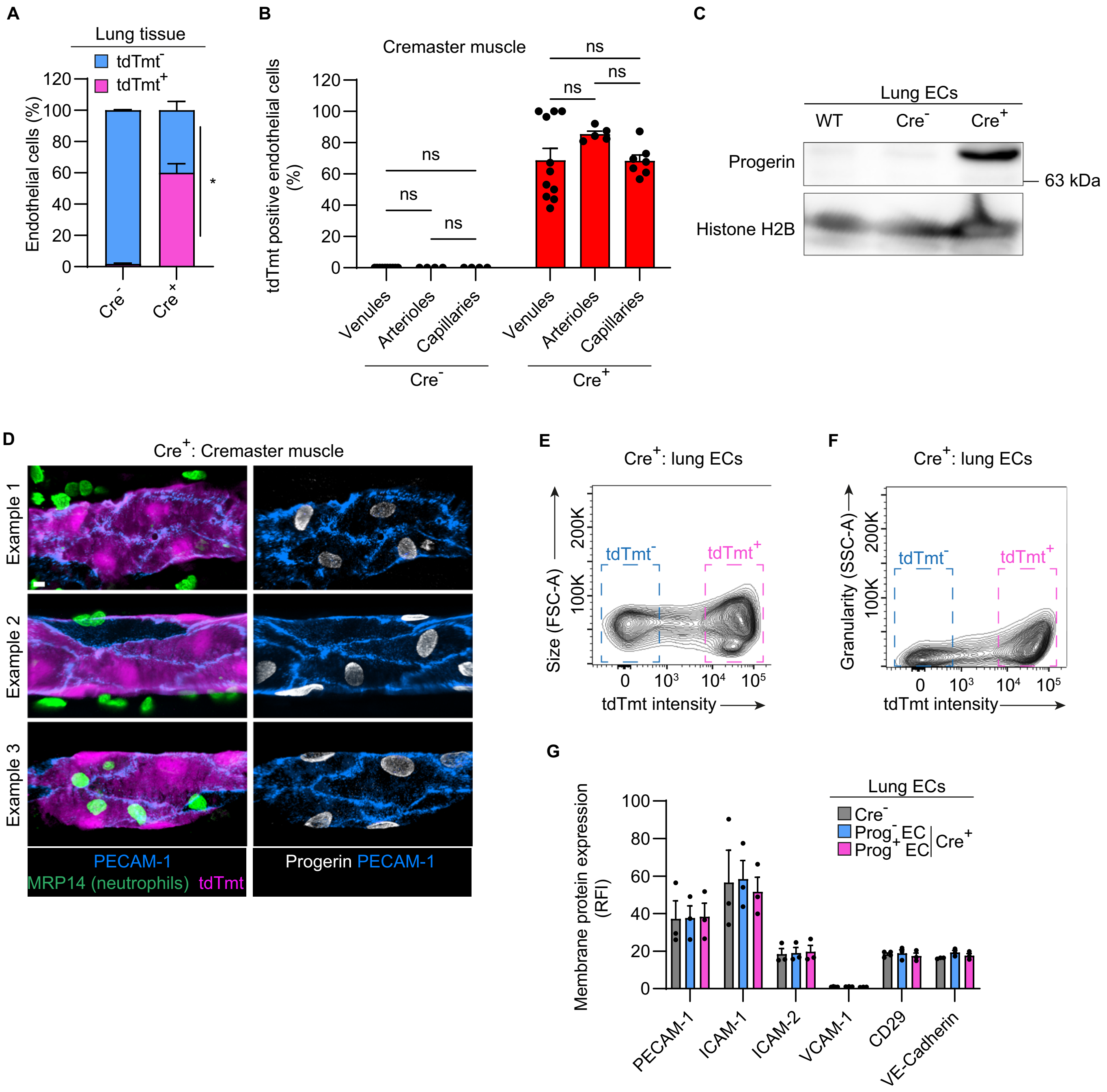

Figure EV1. Cellular and molecular characteristics of tdTmt-progerin-expressing endothelial cells.

(**A, B**) The percentage of tdTomato positive ECs in Cre- and Cre+ mice was quantified in (**A**) lung ECs by flow cytometry ($n = 4$–21 mice/group in 4 independent experiments) and (**B**) cremaster microvasculature ECs by confocal microscopy ($n = 4$–11 mice/group in 4 independent experiments). (**C**) Representative immunoblot for progerin and Histone H2B (loading control) of flow cytometry sorted lung ECs from WT, Cre- and Cre+. (**D**) Representative confocal images of cremaster microvasculature of acutely inflamed (IL-1β) Cre- or Cre+ mice. Anti PECAM-1 mAb was injected i.s. and fixed tissues were immunostained for MRP14 (neutrophils) and progerin. Scale bar: 5 μm. (**E–G**) Lung ECs from Cre+ mice were analyzed by flow cytometry, and (**E, F**) representative plots presented according to tdTomato fluorescence intensity, depicting EC (**E**) size (FSC-B) and (**F**) granularity (SSC-A). (**G**) Surface expression of selected EC proteins quantified and expressed as RFI ($n = 3$–4 mice/group in 6 independent experiments). Data information: (**A, B, G**) Data are mean ± SEM. *$P < 0.05$; ns: not significant. (**A**) Two-tailed paired Student's *t*-test. (**G**) One-way and (**B**) two-way ANOVA test followed by Tukey's post hoc test.

 © The Author(s)

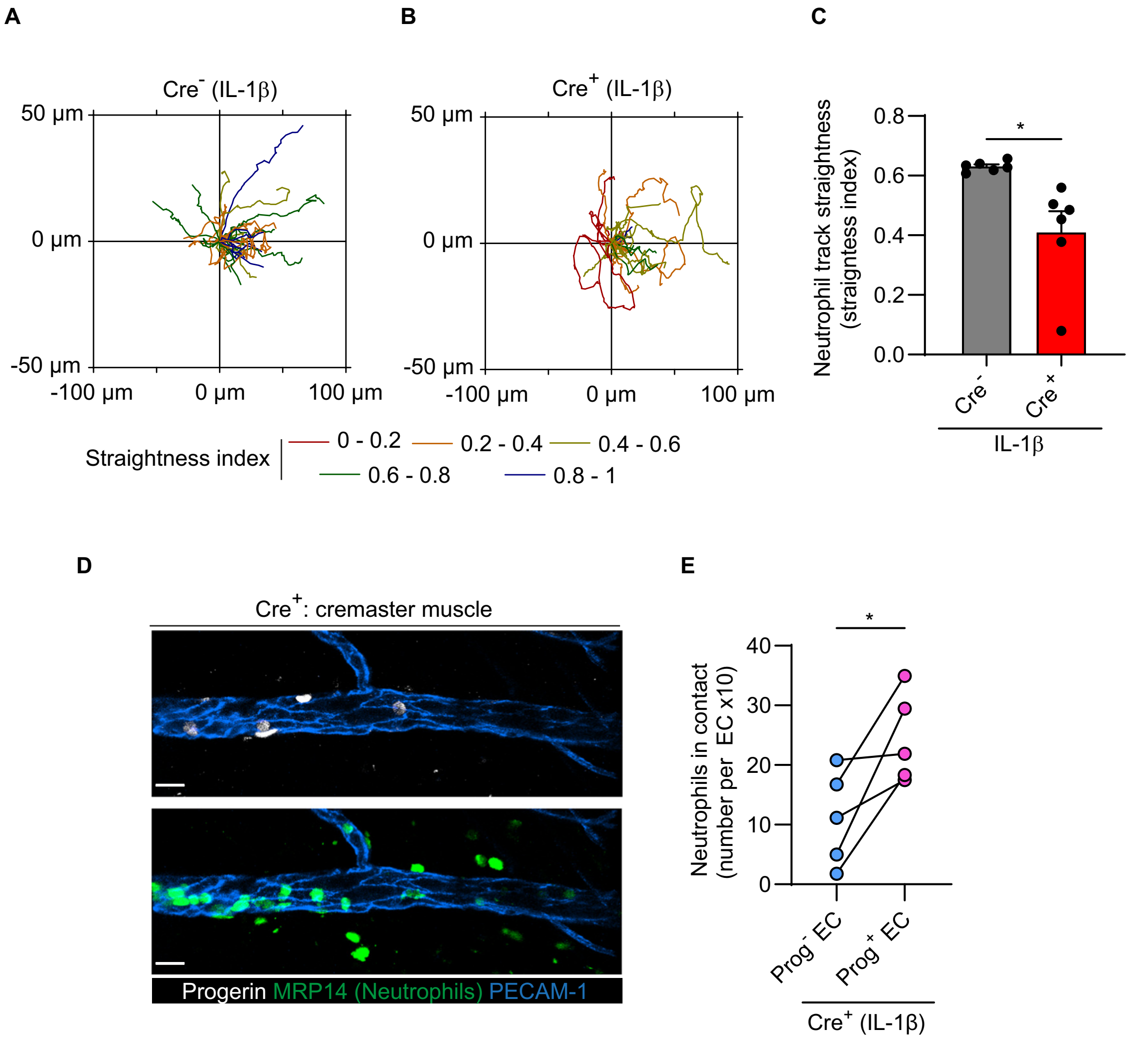

Figure EV2. EC progerin expressing mice exhibit dysregulated luminal neutrophil crawling in IL-1β-stimulated cremasteric venules.

Cremaster microvasculature of *Tie2-Cre;Lmna*$^{LCS/LCS}$*;Rosa26*$^{tdTmt/+}$*;Lyz2-EGFP-ki* Cre$^-$ or Cre$^+$ mice were acutely inflamed (IL-1β) and analyzed by confocal IVM. (**A**, **B**) Crawling profiles of neutrophils on ECs (responses from 28 to 34 neutrophils are displayed). (**C**) Luminal neutrophil track straightness (straightness index; displacement/track length) ($n = 6$ mice/group in 12 independent experiments). (**D**, **E**) Cremaster muscles of *Tie2-Cre;Lmna*$^{LCS/LCS}$*;Rosa26*$^{+/+}$ Cre$^+$ mice were acutely inflamed (IL-1β). (**D**) Fixed cremaster muscles were immunostained for progerin, MRP14 (neutrophils) and PECAM-1 (ECs), and (**E**) number of luminal neutrophils per progerin negative or positive EC in the same vessel segment was quantified ($n = 4$–7 mice/group in 4 independent experiments). Data information: (**C**) Data are mean ± SEM. *$P < 0.05$. Two-tailed unpaired Student's *t*-test. (**E**) *$P < 0.05$. Two-tailed paired Student's *t*-test.

© The Author(s)

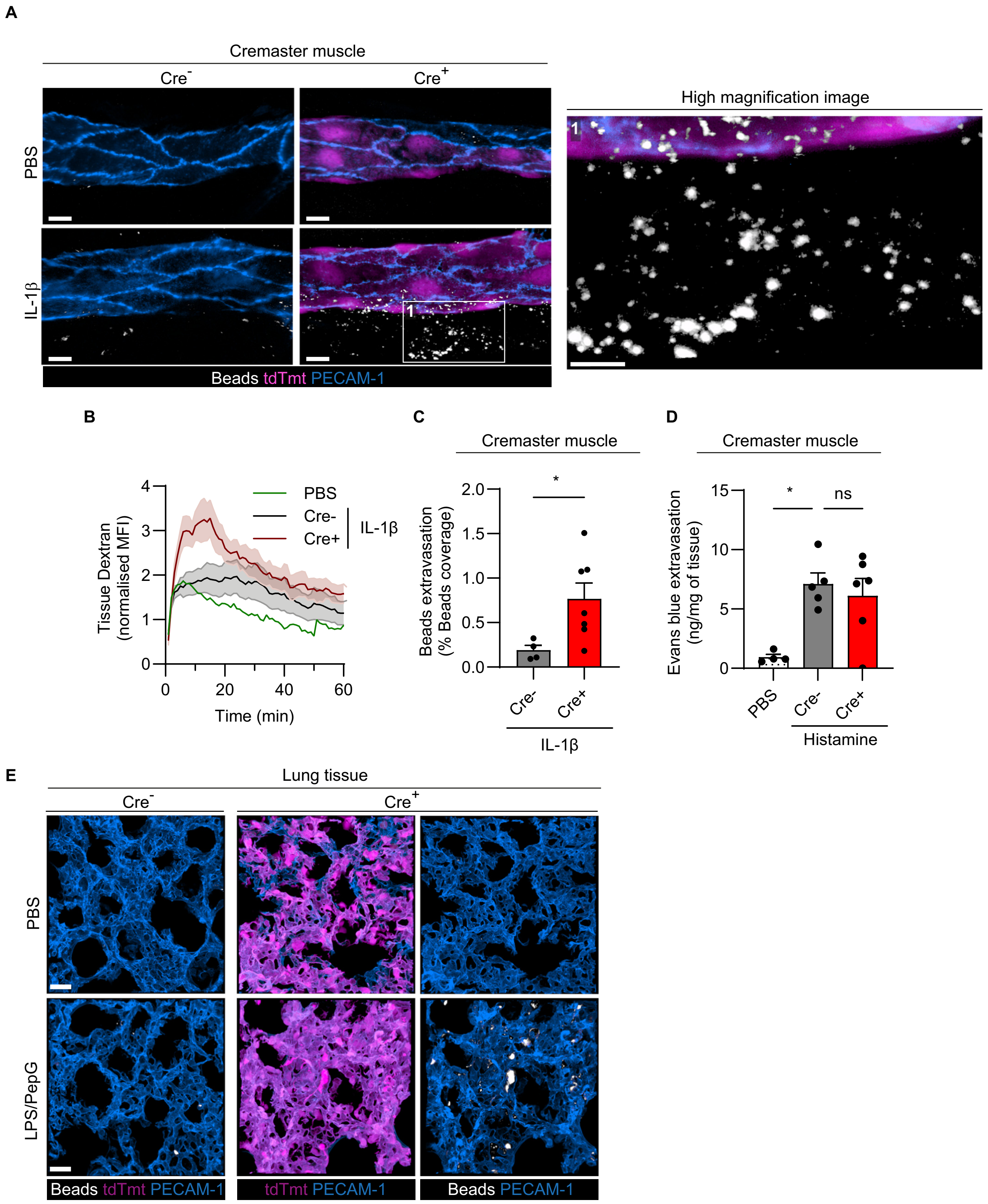
A
Cremaster muscle
Cre-
Cre+
PBS
IL-1β
1
Beads tdTmt PECAM-1
High magnification image
1
B
Tissue Dextran (normalised MFI)
PBS
Cre-
Cre+
IL-1β
Time (min)
C
Cremaster muscle
Beads extravasation (% Beads coverage)
*
Cre-
Cre+
IL-1β
D
Cremaster muscle
Evans blue extravasation (ng/mg of tissue)
*
ns
PBS
Cre-
Cre+
Histamine
E
Lung tissue
Cre-
Cre+
PBS
LPS/PepG
Beads tdTmt PECAM-1
tdTmt PECAM-1
Beads PECAM-1

© The Author(s)

Figure EV3. EC progerin-expressing mice exhibit enhanced microvascular permeability in IL-1β-stimulated cremaster muscles.

(**A**, **B**) Cremaster microvasculature of *Tie2-Cre;Lmna*$^{LCS/LCS}$*;Rosa26*$^{tdTmt/+}$ Cre^{-} or Cre^{+} mice were acutely inflamed with IL-1β or PBS (control) and analyzed by confocal microscopy. Anti-PECAM-1 mAb was injected i.s. and fluorescent beads or 70-kDA dextran were injected i.v. (**A**) Representative confocal images of control (PBS) and stimulated (IL-1β) post-capillary venules illustrating accumulation of extravasated beads in the Cre^{+} sample. White box is magnified and displayed in the right panel. Scale bar: 5 µm. (**B**) Time course of dextran accumulation in the perivascular region of a selected postcapillary venule ($n = 4$–7 mice/group in 18 independent experiments). (**C**) Quantification of microvascular leakage calculated as percentage coverage of extravasated fluorescent beads (percentage coverage) in *Tie2-Cre;Lmna*$^{LCS/LCS}$*;Rosa26*$^{+/+}$ Cre^{-} and Cre^{+} IL-1β-stimulated tissues ($n = 4$–7 mice/group in 4 independent experiments). (**D**) Vascular leakage in the cremaster muscle of *Tie2-Cre;Lmna*$^{LCS/LCS}$*;Rosa26*$^{tdTmt/+}$ Cre^{-} or Cre^{+} mice injected i.s. with PBS, or histamine (30 min) ($n = 4$–7 mice/group in 7 independent experiments). (**E**) Lungs from Cre^{-} and Cre^{+} mice treated i.p. with PBS (control) or LPS/PepG were analyzed by confocal microscopy for vascular leakage (extravasation of i.v. injected fluorescent microbeads). Representative confocal images of lung tissue sections immunostained for PECAM-1 (ECs) illustrate increased bead extravasation (white) in close apposition to tdTmt positive vessel segments of Cre^{+} mice. Scale bar: 30 µm. Data information: (**C**, **D**) Data are mean ± SEM. *$P < 0.05$; ns: not significant. (**C**) Two-tailed unpaired Student's *t*-test and (**D**) one-way ANOVA followed by Tukey's post hoc test.

© The Author(s)

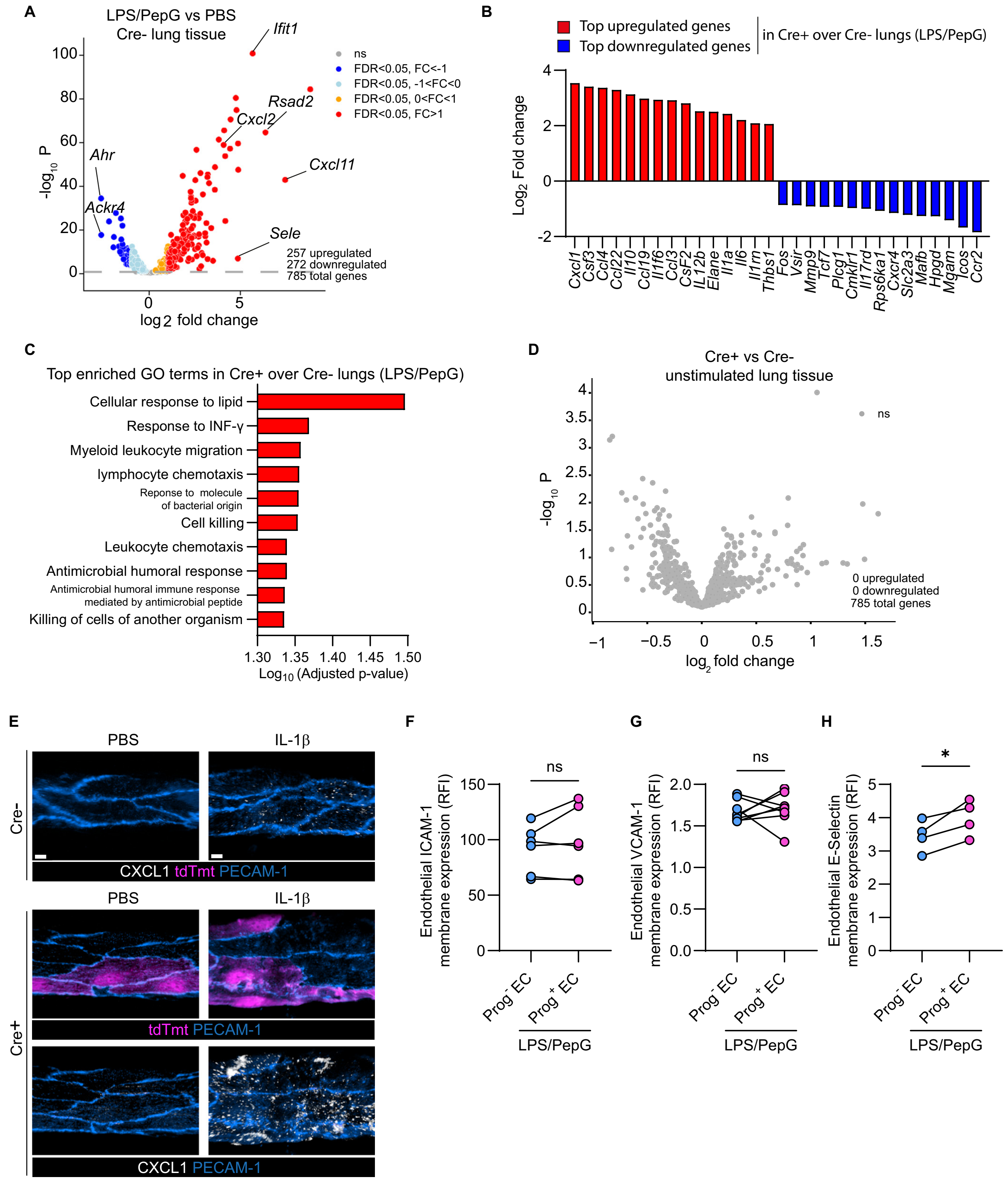
A
LPS/PepG vs PBS
Cre- lung tissue
Ifit1
Rsad2
Cxcl2
Cxcl11
Ahr
Ackr4
Sele
ns
FDR<0.05, FC<-1
FDR<0.05, -1<FC<0
FDR<0.05, 0<FC<1
FDR<0.05, FC>1
257 upregulated
272 downregulated
785 total genes
log 2 fold change
B
Top upregulated genes
Top downregulated genes
in Cre+ over Cre- lungs (LPS/PepG)
Log2 Fold change
C
Top enriched GO terms in Cre+ over Cre- lungs (LPS/PepG)
Cellular response to lipid
Response to INF-γ
Myeloid leukocyte migration
lymphocyte chemotaxis
Reponse to molecule of bacterial origin
Cell killing
Leukocyte chemotaxis
Antimicrobial humoral response
Antimicrobial humoral immune response mediated by antimicrobial peptide
Killing of cells of another organism
Log10 (Adjusted p-value)
D
Cre+ vs Cre-
unstimulated lung tissue
ns
0 upregulated
0 downregulated
785 total genes
log2 fold change
E
PBS
IL-1β
Cre-
Cre+
CXCL1 tdTmt PECAM-1
tdTmt PECAM-1
CXCL1 PECAM-1
F
Endothelial ICAM-1 membrane expression (RFI)
G
Endothelial VCAM-1 membrane expression (RFI)
H
Endothelial E-Selectin membrane expression (RFI)
ns
*
Prog- EC
Prog+ EC
LPS/PepG

 © The Author(s)

Figure EV4. Molecular characteristics of inflamed tissues of EC progerin expressing mice.

(**A–C**) Cre$^-$ and Cre$^+$ mice were stimulated with i.p. PBS or LPS/PepG (2 h) after which tissue samples were analyzed by targeted NanoString transcriptomics. (**A**) Volcano plot of the relative difference in gene expression in whole lung tissue of control or stimulated Cre$^-$ mice as assayed by NanoString (785 genes; $n = 6$ mice/group in 1 experiment). Significantly differentially expressed genes (FDR > 0.05) are color coded as follows: Downregulated genes in dark blue (fold change, FC < −1) and light blue (−1 < FC < 0); upregulated genes in orange (0 < FC < 1) and red (FC > 1). (**B**, **C**) Gene expression profiles in inflamed lung tissues of Cre$^+$ mice, relative to Cre$^-$. (**B**) The 15 most highly upregulated (red) and highly downregulated (blue) genes in Cre$^+$ mice are displayed (785 genes; $n = 3$ mice/group in 1 experiment). (**C**) Gene Ontology (GO) terms enrichment analysis of 785 differentially expressed genes (DEGs) in inflamed lungs of Cre$^+$ mice (scored as $-\log_{10}$ (p-value)). The presented results are based on Fisher's exact test with false discovery rate adjustment. (**D**) Volcano plot of the relative difference in gene expression in whole lung tissue of unstimulated Cre+ vs Cre- mice as assayed by NanoString (785 genes; $n = 3$ mice/group in 1 experiment). (**E**) Representative confocal images of cremaster muscles treated with PBS (control) or IL-1β-stimulated cremaster muscles of Cre$^-$ and Cre$^+$ mice. Tissues were immunostained for PECAM-1 and CXCL1, with tdTomato fluorescence identifying progerin expressing ECs. Scale bar: 5 μm. (**F–H**) Surface expression of (**F**) ICAM-1, (**G**) VCAM-1, and (**H**) E-Selectin, presented as RFI, on progerin negative and positive lung ECs derived from Cre$^+$ mice subjected to LPS/PepG stimulation ($n = 4$–6 mice/group in 6 independent experiments). Data information: (**F–H**) *$P < 0.05$; ns: not significant. Two-tailed paired Student's t-test.

© The Author(s)

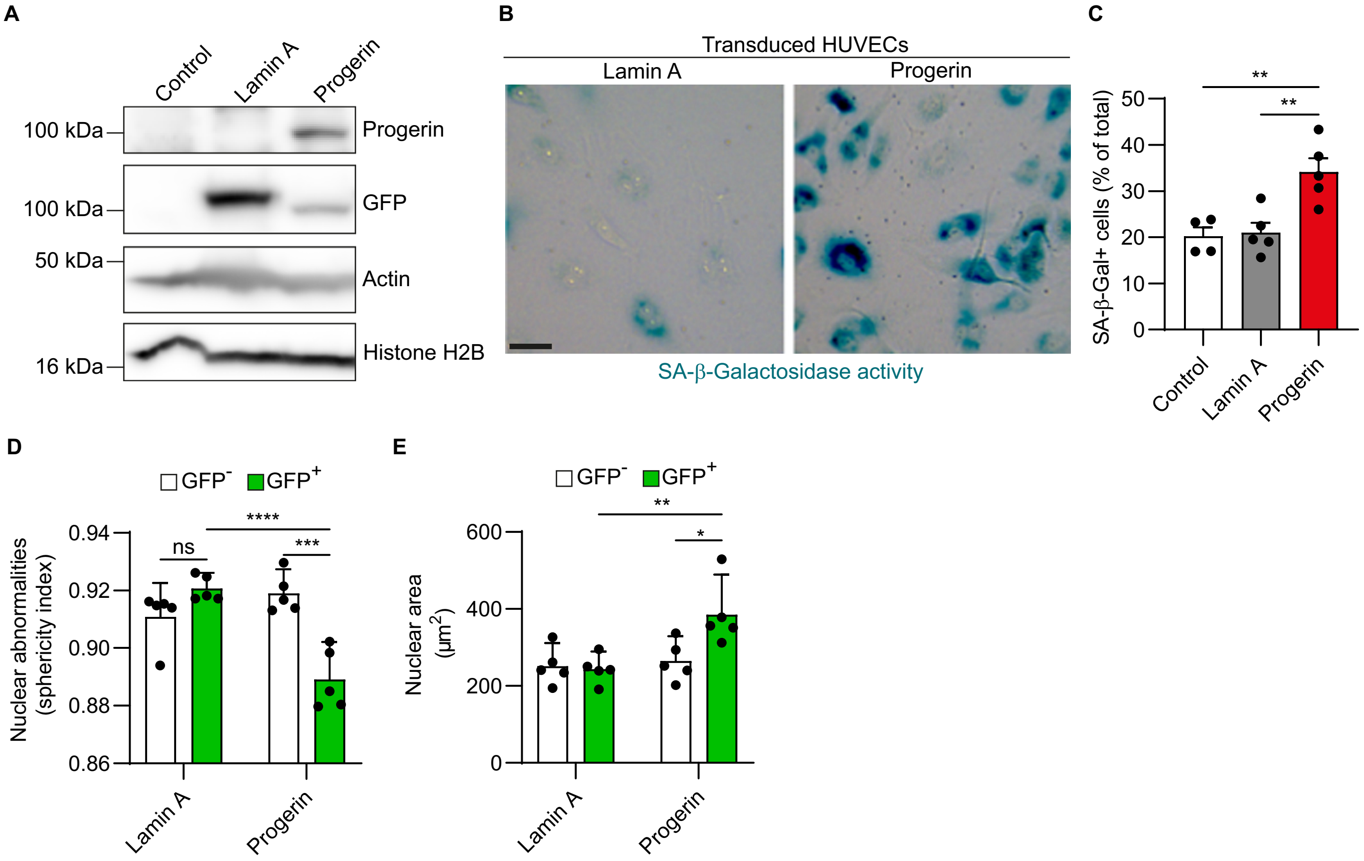

Figure EV5. Progerin expressing HUVECs exhibit cellular senescence and a pro-secretory phenotype.

HUVECs were transduced with GFP-tagged lamin A or GFP-tagged progerin expressing lentiviral constructs. (**A**) Representative immunoblot of control and transduced HUVECs probed for progerin, GFP, β-Actin, and Histone-H2B proteins. (**B**) Representative bright field image of transduced HUVECs assayed for SA-β-Gal activity (blue) and (**C**) its associated quantification ($n = 4$–5 in 5 independent experiments). Scale bar: 40 µm. (**D**, **E**) Non-transduced (GFP-) and transduced (GFP+) HUVECs ($n = 5$ in 5 independent experiments) were quantified for (**D**) nuclear shape abnormalities and (**E**) nuclear size. Data information: (**C–E**) Data are mean ± SEM. *$P < 0.05$; **$P < 0.01$, ***$P < 0.001$; ****$P < 0.0001$; ns: not significant. (**C**) One-way and (**D**, **E**) two-way ANOVA followed by Tukey's post hoc test.

 © The Author(s)
